# Application of 3D-Printed Bioinks in Chronic Wound Healing: A Scoping Review

**DOI:** 10.3390/polym16172456

**Published:** 2024-08-29

**Authors:** Asmaa Y. Abuhamad, Syafira Masri, Nur Izzah Md Fadilah, Mohammed Numan Alamassi, Manira Maarof, Mh Busra Fauzi

**Affiliations:** 1 Department for Tissue Engineering & Regenerative Medicine, Faculty of Medicine, Universiti Kebangsaan Malaysia, Kuala Lumpur 56000, Malaysia; asmaaabuhamad@ukm.edu.my (A.Y.A.); p110574@siswa.ukm.edu.my (S.M.); izzahfadilah@ukm.edu.my (N.I.M.F.); manira@ppukm.ukm.edu.my (M.M.); 2 Advance Bioactive Materials-Cells (Adv-BioMaC) UKM Research Group, Universiti Kebangsaan Malaysia, Bangi 43600, Malaysia; 3 Tissue Engineering Group (TEG), National Orthopaedic Centre of Excellence for Research and Learning (NOCERAL), Department of Orthopaedic Surgery, Faculty of Medicine, Universiti Malaya, Kuala Lumpur 50603, Malaysia; 22114650@siswa.um.edu.my

**Keywords:** chronic wound healing, 3D printing, bioink, cell-laden, regenerative medicine

## Abstract

Chronic wounds, such as diabetic foot ulcers, pressure ulcers, and venous ulcers, pose significant clinical challenges and burden healthcare systems worldwide. The advent of 3D bioprinting technologies offers innovative solutions for enhancing chronic wound care. This scoping review evaluates the applications, methodologies, and effectiveness of 3D-printed bioinks in chronic wound healing, focusing on bioinks incorporating living cells to facilitate wound closure and tissue regeneration. Relevant studies were identified through comprehensive searches in databases, including PubMed, Scopus, and Web of Science databases, following strict inclusion criteria. These studies employ various 3D bioprinting techniques, predominantly extrusion-based, to create bioinks from natural or synthetic polymers. These bioinks are designed to support cell viability, promote angiogenesis, and provide structural integrity to the wound site. Despite these promising results, further research is necessary to optimize bioink formulations and printing parameters for clinical application. Overall, 3D-printed bioinks offer a transformative approach to chronic wound care, providing tailored and efficient solutions. Continued development and refinement of these technologies hold significant promise for improving chronic wound management and patient outcomes.

## 1. Introduction

The integumentary system, the largest organ in the human body, accounts for approximately 15% of total body weight. This vital organ plays a crucial role in the immune system by shielding internal organs from a wide array of pathogens, UV radiation, and physical injuries that pose a threat to human health stability. However, it is highly susceptible to injuries that can compromise the integrity of its layers, from the epidermis to the dermis, resulting in wounds [1,2,3]. Wounds can be classified into two major types: acute and chronic wounds. This classification is based on the healing process and the duration of the healing period. Chronic wounds are defined as damage to the skin layers that do not heal within the expected timeframe of 4 to 8 weeks. Acute wounds, on the other hand, are wounds that heal within a short period due to epidermal/dermal destruction [4]. The Wound Healing Society categorized chronic wounds into four main types: varicose ulcers, venous stasis ulcers, decubitus ulcers, and diabetic ulcers [5]. Chronic wounds have been reported that can impact the quality of life as intensely as heart and kidney diseases. For instance, chronic wounds affect approximately 2% of the population in developed countries, leading to an estimated cost of $25 billion annually in the United States alone [6,7]. The severity of chronic wounds is further underestimated by their high mortality rate, which is now comparable to that of cancer patients. Specifically, diabetes-related foot ulcers and amputations have a 5-year mortality rate ranging from 45% to 95%, making them worse than the mortality rate of many common cancers [8].

Conventional treatments such as sterile gauzes, cotton, surgical plasters, and bandages can only improve the body’s self-healing process [9]. Nevertheless, such traditional practice has limited advantages for chronic or severe wounds, owing to the significant postponement and weakness in the healing process. Yet, the current technological and scientific development has revealed a new sophisticated method that possesses a growing possibility of overcoming chronic wounds. Tissue engineering, as a promising intervention, may offer a biocompatible complex of cells and biomolecules in addition to the other biological components that fabricate the natural human tissues [10,11]. Biofabrication is a developing field of study that involves a process of synthesis of tissues in a tiered designed architecture. The classic biofabrication processes consist of various techniques such as particulate leaching, freeze-drying, electrospinning, and micro-engineering. These processes can produce three-dimensional (3D) structures that are provided with different biomaterials; however, they might be criticized due to the limitations in their reproducibility and flexibility. Previously, tissue engineering has made extensive use of conventional scaffold production methods, including electrospinning, freeze drying, and particle leaching [12]. However, each of these approaches has certain drawbacks. Firstly, electrospinning is a delicate technique that synthesizes 3D structures using electrostatic powers, producing fibers from polymer solutions with nanometer to micrometer diameters and higher surface area [13]. However, certain polymers may emit undesirable or unsafe smells, necessitating procedures to be conducted in closed enclosures with ventilation apertures due to high voltage exposure during fabrication.

Currently, several traditional scaffold fabrication techniques, such as freeze drying, are still being utilized. Briefly, lyophilization is a technique that creates scaffold porosity by forming solvent ice crystals after leaving the composite in the freezer, which is then supplemented with freeze-drying. Ice crystals act as porogens, controlling pore sizes by controlling freezing temperature and polymer weight ratio. This method is beneficial for designing 3D frameworks without organic solvents. The solidified structures undergo sublimation, where water particles evaporate under vacuum and low pressure, resulting in dried 3D scaffolds [13]. Although freeze-drying produces porous structures, uniformity of pore size and distribution is challenging to control, leading to inconsistent scaffold properties. Moreover, some biomaterials may not be suitable for freeze-drying due to potential changes in their physical and chemical properties, potentially impacting their biocompatibility [14].

In addition, in tissue engineering, particulate leaching is a popular method to construct porous scaffolds. However, the fabrication technique has limitations, including difficulty in removing leaching particles and potential cytotoxicity or inflammatory responses if residual particles remain trapped within the scaffold. The solvent-casting particle leaching method achieved pore sizes of 30–300 microns and porosity of 20–50%, with spherical pores and salt particles remaining in the matrix [15]. Thus, bioprinting provides a considerable benefit by minimizing homogeneity issues that might come from post-fabrication cell seeding, as cell placement is performed during fabrication. 

Three-dimensional (3D) bioprinting has rapidly become an innovative fabrication method that can be applied in the development of biomedical procedures such as tissue engineering and various approaches in regenerative medicine [16]. This technology equips researchers with the necessary tools to significantly enhance the construction of fabricated tissues, ensuring high quality, improving reproducibility, and mimicking native tissues [17]. 3D bioprinting differs from traditional additive manufacturing methods by allowing for the creation of customized and precise structures through the adjustment of printing parameters, such as biocompatible materials, temperature, pressure, and speed. The process mainly involves depositing biocompatible materials, either with or without cells, layer-by-layer, to produce predetermined computer-designed structures and functional tissue analogs [18]. This precise control allows for the development of bioscaffolds that can effectively replace damaged skin and enhance its reconstructive qualities, including elasticity and extensibility [19]. Furthermore, this technology reduces the need for multiple surgeries by enabling the precise placement of skin cells and the creation of blood vessel networks, thereby ensuring the long-term viability of the skin tissue [11]. These attributes make 3D bioprinting a promising technology for advancing tissue engineering and regenerative medicine. 

Generally, 3D printing creates a personalized 3D plastic model for a patient, aiding surgeons in planning and performing surgeries. Moreover, 3D bioprinting allows the design of anatomical structures from medical imaging data, as the external size and internal structure of the object closely resemble natural biological tissues [20]. It allows for fine-tuning printing resolution and design, enhancing flexibility and allowing for personalized scaffolding for individual and irregular wound dimensions [21]. Thus, the tool can generate 3D models using Computed Tomography (CT) and Magnetic Resonance Imaging (MRI) data. The data are transformed into 3D digital data using software like mimics (version 14.0), slicers (version Slicer 4), and doctors to create an accurate burn tissue model. Bioink is prepared, and the printed skin is post-processed, maturing in a bioreactor, and tested before being implanted [19].

**Figure 1:**
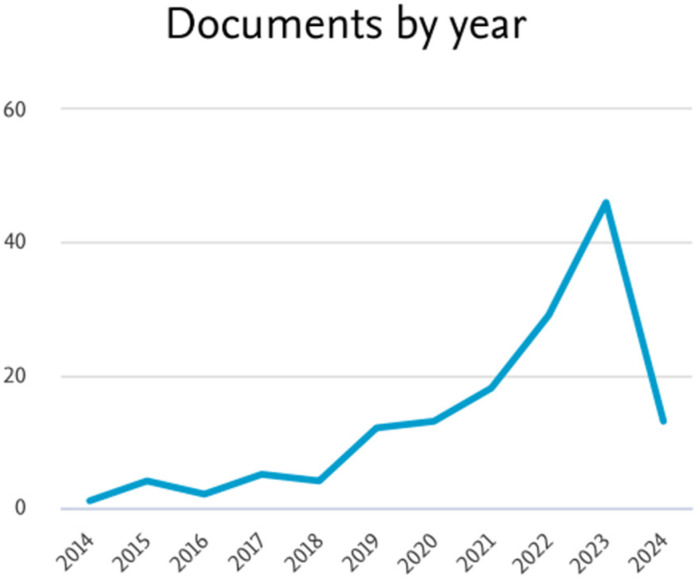
The current trend of publication in the Scopus database for the treatment of chronic wound healing using a 3D-bioprinting method.

As a result, there is an escalating number of publications on the use of 3D bioprinting in the treatment of wound healing (Figure 1). This indicates that the advancement of 3D bioprinting technology has revolutionized tissue engineering, fostering interest in its potential applications in wound healing. Moreover, the biocompatibility, mechanical characteristics, and function of bioprinted tissues have been improved by developments in materials science and bioink formulations, making them more appropriate for use in wound healing applications. The growing interest in 3D bioprinting for wound healing treatment is driven by technological advancements, clinical needs, personalized medical benefits, increased research funding, successful outcomes, and enhanced knowledge dissemination, leading to a growing body of literature exploring its potential in wound care.

**Figure 2:**
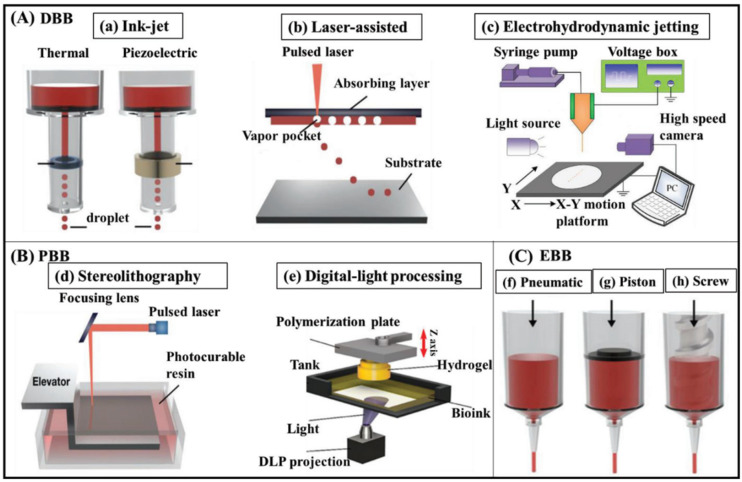
Schematic representation of 3D bioprinting systems. (**A**) Droplet-assisted bioprinting (DBB) includes (**a**) inkjet, (**b**) laser-assisted, and (**c**) electrohydrodynamic jetting. (**B**) Photocuring-based bioprinting (PBB) includes (**d**) stereolithography and (**e**) digital light processing. (**C**) extrusion-based bioprinting (EBB) includes (**f**) pneumatic, (**g**) piston, and (h) screw. Adapted from [43].

3D bioprinting techniques can be classified into three main types: droplet-based bioprinting (DBB), photocuring-based bioprinting (PBB), and extrusion-based bioprinting (EBB) (Figure 2). Each type has unique advantages and disadvantages (Table 1) [22,23]. DBB encompasses inkjet, laser-assisted, and electrohydrodynamic jetting technologies. Inkjet bioprinting offers high precision and minimal bioink waste; however, its capabilities are limited by its small apertures and nonporous structures [24]. Inkjet printing produces droplets of picoliter size, allowing for the precise digitization of microlevel biological elements. Its 50 μm nozzle diameter, similar to that of cells, makes it suitable for cell and single-cell printing, enhancing the precision of digitized patterns [25].

Laser-assisted bioprinting achieves high-resolution, non-contact printing with high cell survival rates but is hindered by slow gelation [26]. LAB bioprinting has superior printing precision and resolution than nozzle-based technologies, reaching micron levels under optimum conditions and allowing for the separation of single cells or cell aggregations from bioink at high concentrations. Moreover, this printing method is widely used to produce biological materials with high cell viability, density, and bioprinting resolution of 20–30 μm. Electrohydrodynamic jetting uses an electric field to eject droplets, preventing excessive cell pressure; however, it encounters material propagation issues with large droplet sizes [27]. 

Meanwhile, PBBs such as stereolithography (SLA) and digital light processing (DLP) use light to solidify photosensitive polymers, avoiding issues related to nozzles and shear stress. SLA can be used to create high-resolution porous scaffolds with good cell viability. The only restriction for SLA is the requirement of using a transparent liquid to enable light to penetrate the substance and ensure consistent crosslinking. SLA offers a significant advantage in improving printing resolution by reducing the laser spot size up to 10 mm, in addition to influencing the printing speed, direction, and volume [28]. Consequently, the cell concentration in the bioink is restricted to a maximum of around 108 cells/mL [28,29]. 

DLP has the capability to solidify entire layers rather than using point-by-point photocuring. It starts by printing the bottom layer first and then successively adding each new layer on top of the previous one, resulting in a smooth and sturdy structure. However, this method has the drawback of only utilizing photosensitive materials [30]. EBB involves the precise deposition of continuous bioink filaments using piston, screw, or pneumatic methods. This approach is particularly suitable for various bioinks, including high-viscosity hydrogels, due to its ability to provide better mechanical strength and faster printing speed compared to drop-on-demand inkjet bioprinting but has lower resolution compared to DBB. Screw-driven printers are particularly effective for creating stable structures from high-viscosity bioinks. On the other hand, pneumatic printers are efficient in handling shear-thinning hydrogels, ensuring the precise and consistent deposition of bioink [31,32]. However, bioinks typically have limited resolution, ranging from hundreds of micrometers to millimeters, due to the minimized shear stresses at the dispenser tip and nozzle diameter to prevent cell damage [33].

**Table 1:** Summary of Bioprinting Techniques.

Type of Bioprinting	Sub-Type	Definitions	Advantages	Limitations	Applications	References
Droplet-based Bioprinting (DBB)	Inkjet-based bioprinting	Inkjet-based bioprinting is a method that uses droplets to create tissue and organ constructs on a substrate.	low cost, high precision, fast fabrication speed, high resolution, and high throughput	High-viscosity ink has poor printability, small inkjet apertures, easy clogging, and high shear stress.	Fabrication of scaffold for tissue engineering	[19,34,35]
	Laser-assisted bioprinting (LAB)	LAB uses the Laser-Induced Forward Transfer (LIFT) technique, where a pulsed laser beam is focused onto a bioink-coated substrate, creating a high-pressure bubble.	high resolution, no nozzle clogging, and non-contact printing	Thermal damage and slow gelation limit high-throughput printing and expensive equipment	Fabrication of scaffold for tissue engineering	[34,36]
	Electrohydrodynamic Jetting (EHDJ)	EHDJ uses high voltage to create an electric field that shapes bioink into a cone shape at the nozzle tip.	Preventing excessive pressure, protecting cell viability, high cell concentration, and high weight-to-volume ratio in bioink	Affecting cell viability and limited material propagation for larger droplet sizes (>400 µm)	Fabrication of scaffold for tissue engineering	[27,37]
Photocuring-based Bioprinting (PBB)	stereolithography	Stereolithography (SLA) is a photochemical additive manufacturing technique that creates three-dimensional objects by curing a liquid photopolymer resin with a focused UV laser or light source.	High resolution, rapid printing with high cell viability (>85%), no shear stress on cells, and free clogging process	Requires transparent liquids for uniform crosslinking, cell contamination, and Restricted maximum cell density	Fabrication of scaffold for tissue engineering	[36,38,39]
	Digital Light Processing (DLP)	DLP uses a digital light source to cure photosensitive polymers layer-by-layer using a dynamic mask that projects the desired pattern onto the bioink.	High-speed printing, production of complex structures without increasing time, Smoother 3D structures, and improving mechanical strength	Less suitable for applications requiring varying layer thicknesses, require complex setup and software for mask design and light pattern transmission	Fabrication of scaffold for tissue engineering	[30,40]
Extrusion-based bioprinting (EBB)	Piston, Screw, Pneumatic-driven	Extrusion-based bioprinting techniques utilize the usage of a nozzle or syringe to extrude the bioink via piston, pressure, and screw-driven approach.	High-viscosity bioinks - Versatile for different bioink types - Better printing speed and mechanical strength compared to DBB	Limited printing resolution limits cell patterning and organization, and High shear stress	Fabrication of scaffold for tissue engineering	[24,41,42]

Each 3D bioprinting technique is designed for specific tissue engineering and regenerative medicine applications, balancing precision, cell viability, and material compatibility. By understanding the capabilities of each technique, researchers can choose a specific technique to achieve the desired results for various applications.

Bioink, a mixture of cells, biomaterials, and biologically active elements, is crucial in 3D bioprinting [44]. This review focuses on bioinks with living cells, excluding those with polymers without embedded cells or seeded post-printing. In general, the composition of bioinks consists of natural, synthetic biomaterials or a combination of both [45]. Natural biomaterials such as gelatin, alginate, collagen, hyaluronic acid, chitosan, and decellularized extracellular matrix (dECM) offer advantages such as biocompatibility, biodegradability and the ability to replicate the structure of the extracellular matrix (ECM) [46]. These attributes are crucial for establishing a conducive microenvironment for cell growth and function. On the other hand, synthetic polymers like polyethylene glycol (PEG), polylactic acid (PLA), and ε-caprolactone have been employed to mimic the mechanical characteristics and degradation details of biological tissues, facilitate drug elimination, and respond to physical conditions such as PH and thermal changes [47].

Skin tissue engineering has shifted significantly since three-dimensional bioprinting emerged. This technology offers a promising treatment strategy that ensures the possibility of an automated, well-designed complex biological structure mimicking native tissues and overcoming conventional techniques’ limitations [48]. A few studies using different electronic databases have discussed the application of 3D bioprinting technology in the field of chronic wounds. To our knowledge, this scoping review is among the first to comprehensively evaluate the application of 3D bioprinting in chronic wound healing, with a specific focus on bioinks incorporating living cells. This scoping review aims to identify the current advances in 3D bioprinting for chronic wounds and address the following questions: (i) How has 3D bioprinting technology been applied in chronic wounds? (ii) Which types of biomaterials and cells are used for 3D bioprinting?

## 2. Methods

### 2.1. Search Strategy

This review adheres to the methodological framework of the Joanna Briggs Institute guidelines for scoping reviews and conforms to the Reporting Items for Systematic Reviews and Meta-Analyses extension for Scoping Review (PRISMA-ScR) guidelines [49,50]. This review is driven by two primary research questions: (i) How has 3D bioprinting technology been applied in the management of chronic wounds? This question seeks to explore the various applications of 3D bioprinting technologies in the treatment and management of chronic wounds by examining methodologies and innovations in the field. (ii) What types of biomaterials and cells are used in 3D bioprinting for chronic wound healing? This question aims to identify the different biomaterials and cellular components used in 3D bioprinting processes and assess their effectiveness in promoting the healing of chronic wounds. These questions frame the review’s scope, guiding the systematic collection and analysis of relevant data to provide comprehensive insights into the current state and advancements of 3D bioprinting technologies in chronic wound healing applications.

A comprehensive literature search was performed through May 2024 using the PubMed, Scopus, and Web of Science databases. The search strategy employed the following terms: (“Diabetic foot ulcer*” OR “Pressure ulcer*” OR “Venous ulcer*” OR “Chronic wound*” OR “Non-healing wound*” OR “Persistent wound*” OR “Nonhealing wound*” OR “Chronic ulcer*” OR “Non-healing ulcer*” OR “diabetic wound*”) AND (“3D bioprint*” OR “3D print*” OR “3D-print*” OR “3D-bio-print*” OR “bioprinting” OR “3D cell printing” OR “3D scaffold” OR “3D prototyping” OR “Three-dimensional bioprint*” OR “Three dimensional printing”) AND (“skin” OR “derm*” OR “epiderm*” OR “cutaneous” OR “integument*” OR “cutis”). The search was limited to articles published in English between 2019 and 2024. In brief, the search strategy integrated terms related to “3D bioprinting”, “chronic wounds”, and “skin”, ensuring a broad capture of relevant studies. These terms were applied to both the title and abstract of the articles.

### 2.2. Study Selection

The initial screening was based on titles and abstracts and was conducted independently by two reviewers (A.Y.A and S.M). Initial screening was conducted via titles and abstracts using Rayyan software (version Rayyan 1.4.4) [51]. The full texts of potentially eligible studies were then retrieved manually for a more detailed evaluation according to the predefined inclusion and exclusion criteria. Disagreements between the reviewers were resolved through discussion or, if necessary, adjudication by a third reviewer (N.I.M.F).

The inclusion criteria for the studies were defined based on the Participant/Population (P): cell-laden 3D-bioprinted constructs; Concept (C): interventions aimed at enhancing chronic wound healing using bioinks that incorporate living cells, potentially in combination with biomaterials and/or growth factors applied before or during printing; Context (C): application of 3D bioprinting in tissue-engineered strategies specifically targeted at treating chronic wounds. The inclusion criteria were as follows: original research articles involving 3D bioprinting technologies applied directly to chronic wound management, utilizing bioinks containing living cells, and reporting measurable outcomes related to wound healing. Exclusion criteria included review articles, studies not focusing on chronic wounds (such as acute wound healing or non-wound related applications), and studies not using 3D bioprinting technologies or living cells.

### 2.3. Data Extraction and Analysis

Data extraction was performed by the first reviewer and verified by the second reviewer to ensure accuracy and alignment with the research questions. The synthesized data included publication details (authors, year of publication, and country), study design (in vitro, in vivo, and clinical trials), types of 3D bioprinting techniques and parameters, materials and cells used, and specific outcomes related to chronic wound healing. The results were tabulated to highlight the key findings related to the application of 3D bioprinting in chronic wound management and to address the complexity and diversity of the technology and its outcomes.

### 2.4. Data Charting and Synthesis

Data extraction focused on methodologies, bioink components, wound types addressed, and therapeutic outcomes. The results were synthesized to map the current landscape and evaluate the applicability of the findings.

## 3. Results

### 3.1. Study Selection 

**Figure 3:**
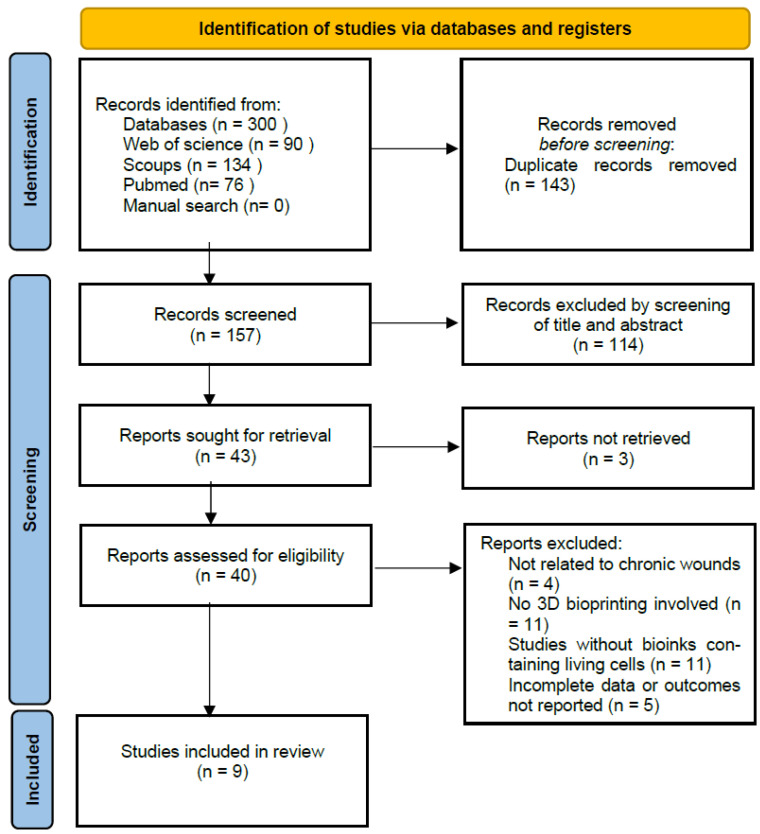
PRISMA flow diagram illustrating the results of the search strategy.

The systematic search and study selection process for our review is illustrated in Figure 1. Initially, a comprehensive search of various databases yielded a total of 300 records, accessed on the 9th of May 2024. The databases included PubMed (n = 76), Web of Science (n = 90), and Scopus (n = 134). From these, we removed 143 duplicate records, resulting in 157 records for initial screening that were screened based on the title and abstract. Of the screened records, 114 were excluded due to being review articles or other publication types that were not original research articles, such as editorials, commentaries, and conferences, or not related to the topic scope. This led to a detailed evaluation of 43 full-text articles to determine their eligibility based on specific inclusion and exclusion criteria. Reasons for exclusion included studies that were not accessible (3), not relevant to the chronic wound topic (4), studies that did not use 3D bioprinting technologies (11), studies not involving bioinks containing living cells (11), and studies not meeting other specific inclusion criteria (5). After screening, 43 full-text articles were assessed for eligibility, of which 31 were excluded. Following this assessment, nine studies were deemed appropriate and were included in the review. These studies primarily focused on the application of 3D printed bioinks in chronic wound healing, adhering to our rigorous inclusion criteria of using living cells within the bioinks and relevance to chronic wound management. The diagram provides a comprehensive overview of the filtering process, ensuring the transparency and reproducibility of our research methodology, as outlined in Figure 3.

### 3.2. Characteristics of Included Studies

This section summarizes the findings of the reviewed studies, focusing on the 3D bioprinting techniques, bioink compositions and characteristics, applications, and models used in these studies. The results are organized into five comprehensive tables, followed by detailed descriptions for each table.

The studies included in this review highlight the versatility and potential of 3D bioprinting techniques and bioink compositions for wound healing applications. These studies varied in study design, types of 3D bioprinting techniques, bioink components, and specific outcomes related to chronic wound healing. The studies span various countries, predominantly employing the extrusion-based 3D bioprinting technique with diverse bioink formulations aimed at treating chronic wounds, such as diabetic ulcers. The reviewed studies, summarized in Table 2, highlight various approaches for utilizing cell-laden bioinks in 3D bioprinting for chronic wound healing. Schmitt et al. (2021) [52] demonstrated sustained cytokine release and high cell viability using methacrylated collagen (CMA) and microfat for chronic wounds. Manso et al. (2023) [53] reported accelerated wound healing and increased mast cell presence in chronic diabetic ulcers using alginate hydrogel embedded with mesenchymal stem cells (MSCs). Bajuri et al. (2023) [54] achieved significant wound size reduction and 70% complete healing within 12 weeks in diabetic foot ulcer (DFU) patients using autologous adipose tissue and fibrin gel. Ullah et al. (2023) [55] utilized a synthetic bioink (PEO-CS-PMMA with nicotinamide) for skin tissue regeneration, noting improved mechanical properties and increased cell viability. Xia et al. (2022) [56] incorporated curcumin into a GelMA scaffold for diabetic wound healing, resulting in reduced reactive oxygen species (ROS), decreased apoptosis, and improved cell survival. Ma et al. (2021) [57] combined strontium silicate with a gellan gum (GG), sodium alginate (AG), and methyl cellulose (MC) bioink, demonstrating enhanced cell proliferation and blood vessel formation in acute and chronic wounds. Masri et al. conducted two studies, one in 2024 [58] and another in 2023 [59], using a gelatin and polyvinyl alcohol (PVA) bioink, showing improved printability, cell viability, and wound healing rates, with additional benefits of reduced inflammation and optimal water vapor transmission rate (WVTR). Lastly, Albanna et al. (2019) [60] developed a mobile 3D bioprinter for customized in situ grafts using dermal fibroblasts, epidermal keratinocytes, and fibrin gel, with notable healthy wound closure. 

The summarized studies collectively underscore the promising role of extrusion-based 3D bioprinting using various bioinks in improving chronic wound healing. The studies originate from a diverse range of countries, with the USA (Schmitt et al., 2021 [52]; Albanna et al., 2019) [60] and Malaysia (Bajuri et al., 2023 [54]; Masri et al., 2024 [58]; Masri et al., 2023) [59] contributing significantly. Other countries include Brazil (Manso et al., 2023) [53], Pakistan (Ullah et al., 2023) [55], and China (Xia et al., 2022 [56]; Ma et al., 2021) [57]. This geographical distribution reflects the global interest in the development of advanced bioprinting techniques for chronic wound management. The temporal trend shows an increase in publications from 2019 to 2024, highlighting the rapid development and adoption of 3D bioprinting technologies. This distribution indicates a growing interest and investment in the application of 3D bioprinting technologies for chronic wound healing in recent years.

The study designs vary, including in vitro, in vivo, and single-arm pilot studies. The majority of studies employ in vitro and in vivo methodologies, with in vitro studies accounting for three (Schmitt et al., 2021 [52]; Masri et al., 2024 [58]; Masri et al., 2023 [59]) and in vivo studies also accounting for three (Manso et al., 2023 [53]; Ullah et al., 2023 [55]; Xia et al., 2022) [56]. Two studies combined in vitro and in vivo approaches (Xia et al., 2022 [56]; Ma et al., 2021 [57]), and one was a single-arm pilot study (Bajuri et al., 2023) [54]. This distribution indicates a balanced approach between laboratory and real-world applications, which enhances the robustness of the findings.

Extrusion-based 3D bioprinting is the predominant technique used in all studies, reflecting its popularity and suitability for bioink applications. The single exception is Albanna et al. (2019) [60], which employed mobile in situ skin inkjet-based bioprinting with integrated imaging technology (Supplementary Figure S1). This consistent use of extrusion techniques suggests a trend toward methods that offer precision and control in bioink deposition. 

The bioink compositions vary widely, demonstrating innovation and diversity in material selection. Commonly used polymers mixed with cells to form bioinks include methacrylated collagen (Schmitt et al., 2021) [52], alginate hydrogel (Manso et al., 2023) [53], fibrin gel (Bajuri et al., 2023 [54]; Albanna et al., 2019) [60], and combinations such as PEO-CS-PMMA and nicotinamide (Ullah et al., 2023) [55], GelMA and curcumin (Xia et al., 2022) [56], and SS-GAM (Ma et al., 2021) [57]. Gelatin, PVA, and genipin were used in two studies by Masri et al. (2024 [58]; 2023 [59]). This diversity reflects ongoing experimentation and optimization in the field to identify the most effective formulations.

The key outcomes of these studies included high cell viability and accelerated wound healing, as noted in the majority of the studies. These outcomes indicate that the bioinks used supported cell survival and function effectively and enhanced the wound-healing process. Other notable outcomes were sustained cytokine release, increased mast cells, improved regenerative profile, significant wound size reduction, no adverse events, high reproducibility, improved mechanical properties, reduced reactive oxygen species (ROS), decreased apoptosis, improved ADSC survival, blood vessel formation, graft-host integration, enhanced cell proliferation, improved printability, improved rheological properties, reduced inflammation, optimal water vapor transmission rate (WVTR), and a slow biodegradation rate.

Overall, the included studies collectively underscore the significant potential of 3D bioprinting and bioinks in enhancing wound healing processes, with promising outcomes that suggest broader clinical applications in regenerative medicine. Further research and standardized methodologies could enhance the reproducibility and efficacy of these promising bioprinting technologies.

### 3.3. Characteristics of the 3D Bioprinting Techniques

The studies examined reveal diverse 3D bioprinting techniques and parameters, showcasing the adaptability and specificity of bioprinting methods tailored to various bioink compositions and therapeutic needs. Commonly used bioprinting techniques include extrusion-based 3D bioprinting techniques. The effectiveness of 3D bioprinting in chronic wound healing relies heavily on the selection of appropriate bioinks, bioprinter models, and precise control of printing parameters. Table 3 provides a detailed summary of the characteristics of the 3D bioprinting techniques used in the included studies. 

Schmitt et al. (2021) [52] used REGEMAT3D with UV crosslinking at room temperature, achieving high print fidelity and stability. Manso et al. (2023) [53] did not specify their bioprinter model but highlighted the use of alginate hydrogel for MSCs. Bajuri et al. (2023) [54] utilized the Dr. INVIVO clean chamber 3D bioprinter with fibrin gel crosslinking, tailored according to wound size. Ullah et al. (2023) [55] focused on thermal initiation and chemical crosslinking with carbohydrazide for their fibroblast-PEO-CS-PMMA bioink, operating at 37 °C. Xia et al. (2022) [56] used the BioArchitect Pro with blue light crosslinking for their GelMA-Curcumin bioink, achieving a circular mesh structure. Ma et al. (2021) [57] employed BioScaffolder 3.2 with ionic crosslinking, producing hexagonal prism structures. Both Masri et al. (2024) [58] and Masri et al. (2023) [59] used Biogens XI for their HDF-Gelatin-PVA bioinks, achieving high print fidelity and stability through chemical crosslinking with genipin. 

The cell-laden bioinks used across the studies included natural polymers, synthetic polymers, and hybrid polymers that combined natural and synthetic components. Notably, natural polymers, such as methacrylated collagen (CMA), alginate hydrogel, fibrin gel, gelatin methacryloyl (GelMA), and a combination of gellan gum (GG), sodium alginate (AG), and methyl cellulose (MC), were predominantly used. Synthetic polymers like PEO-CS-PMMA were also employed, reflecting the ongoing exploration of various material properties to enhance bioprinting outcomes. Hybrid bioinks combining gelatin and polyvinyl alcohol (PVA) were used in some studies to leverage the advantages of both natural and synthetic materials.

A variety of 3D bioprinter models were used, including REGEMAT3D, Dr. INVIVO clean chamber 3D bioprinter, BioArchitect Pro, BioScaffolder 3.2, and Biogens XI, while Manso et al. (2023) [53] employed a model developed by In Situ Cell Therapy. Albanna et al. (2019) [60] employed a custom-designed mobile bioprinter. Extrusion-based bioprinting has emerged as the predominant technique, highlighting its robustness and versatility. However, one study by Albanna et al. (2019) [60] utilized an inkjet-based technique, reflecting an alternative approach for specific applications.

Nozzle sizes varied from 0.21 mm to 0.965 mm, with most studies specifying sizes around 0.25 mm to 0.3 mm, indicating the customization required for different bioink formulations and applications. The choice of nozzle size is crucial for achieving the desired resolution and structural integrity. Printing parameters, such as speed and pressure, are critical for maintaining the structural integrity and cell viability of the printed constructs. The printing speeds ranged from 5 mm/s to 9417.45 mm/s, while printing pressures ranged from 20 kPa to 280 kPa. For instance, Xia et al. (2022) [56] utilized a printing speed of 5 mm/s and pressure of 200 kPa for their GelMA bioink, optimizing the process for their specific application without damaging the cells.

Crosslinking methods are essential for stabilizing printed structures and include UV crosslinking, chemical crosslinking with thrombin or genipin, thermal initiation with chemical crosslinking using carbohydrazide (CH), blue light crosslinking, and ionic crosslinking with calcium chloride (CaCl_2_). These methods were chosen based on the bioink composition and the desired mechanical properties of the final printed constructs. The printing environments were controlled for temperature, with some studies specifying room temperature conditions, while others maintained specific temperatures for the nozzle and platform, such as 20 °C for the nozzle and 18 °C for the platform in the study by Xia et al., 2022 [56]. Albanna et al. (2019) [60] utilized a mobile operating room setup. These conditions are critical for maintaining cell viability and ensuring successful bioprinting.

The shapes of the printed constructs were designed to mimic native tissue structures, including cubes, circular mesh structures, hexagonal prisms, grid-like patterns, and tailored wound topographies. These shapes are crucial for facilitating cellular nutrient uptake and biochemical signaling, thereby enhancing the functionality of bioprinted tissues. Crosslink times also varied significantly, from as short as 20–30 s for blue light crosslinking to as long as 10 h for thermal and chemical crosslinking, reflecting the diverse requirements of each bioink formulation.

The detailed analysis of 3D bioprinting techniques in these studies underscores the versatility and adaptability of extrusion-based bioprinting for chronic wound healing applications. The variability in bioink compositions, bioprinter models, printing parameters, and crosslinking methods highlights the need for continued research to optimize these factors to improve clinical outcomes. This comprehensive overview serves as a foundation for further research and standardization efforts aimed at maximizing the efficacy of 3D bioprinting technologies in biomedical applications.

### 3.4. Bioink Composition and Characteristics

Next, the reviewed studies demonstrate a diverse range of bioink compositions and their associated properties, which are crucial for the successful application of 3D bioprinting in chronic wound healing. Table 4 provides a detailed summary of the cell-laden bioink formulations, cell types, preparation methods, and key properties of the bioinks used in the studies.

These studies utilized a variety of cell-laden bioinks, combining different cell types to enhance the therapeutic potential of bioprinted constructs. Notably, adipose stromal cells (ASCs), endothelial progenitor cells (EPCs), mesenchymal stem cells (MSCs), human dermal fibroblasts (HDFs), adipose-derived stem cells (ADSCs), human umbilical vascular endothelial cells (HUVECs), murine umbilical vein endothelial cells (MUVECs), and BALB/3T3 fibroblasts. These cell types were selected for their regenerative capabilities and compatibility with bioink materials.

Cell densities varied significantly across the studies, ranging from 1 × 10^4^ to 7.5 × 10^6^ cells/mL. High cell viability was a common outcome, with several studies reporting viability rates of over 90%. Ullah et al. (2023) [55] achieved a cell viability of 92% with their fibroblast-PEO-CS-PMMA bioink, indicating a robust biocompatibility and the ability of the bioink to support cell survival during and after the bioprinting process. Similarly, Masri et al. (2023 [59], 2024 [58]) reported cell viabilities greater than 90% for their HDF-Gelatin-PVA bioinks, reinforcing the potential for these bioinks in clinical applications.

Bioink preparation methods include mixing, free radical copolymerization, and chemical crosslinking. For example, Schmitt et al. (2021) [52] and Manso et al. (2023) [53] employed mixing, while Ullah et al. (2023) [55] used free radical copolymerization, which exhibited high thermal stability and viscosity. Xia et al. (2022) [56] mixed ADSCs with GelMA and curcumin, followed by blue light crosslinking. Ma et al. (2021) [57] utilized mixing and ionic crosslinking, and both studies by Masri et al. (2023 [59], 2024 [58]) employed chemical crosslinking with genipin to enhance the mechanical properties and porosity of their HDF-Gelatin-PVA bioink. Albanna et al. (2019) [60] printed a fibrinogen/collagen solution directly onto the wound, followed by thrombin printing to form the gel. 

The properties of the bioinks were tailored to meet the specific requirements of different wound types. High viscosity, optimal water vapor transmission rate (WVTR), and excellent interconnected porosity were some of the key characteristics reported. For instance, Xia et al., 2022 [56], highlighted the high porosity and suitable mechanical properties of their GelMA-Curcumin bioink. High porosity allows for effective nutrient exchange and waste removal, which are critical for cell survival and proliferation. Ullah et al. (2023) [55] reported a viscosity range of 500–550 Pa.s and thermal stability of their fibroblast-PEO-CS-PMMA bioink, crucial for maintaining the structural integrity of the printed constructs Ma et al. (2021) [57] noted high viscosity, cell adhesion, and excellent proliferation. Both studies by Masri et al. (2024 [58], 2023 [59]) reported high viscosity, optimal water vapor transmission rate (WVTR), and excellent interconnected porosity. Albanna et al. (2019) [60] emphasized suitable support for cellular viability and rapid crosslinking. The properties of these bioinks not only support cell viability and proliferation but also ensure the mechanical stability necessary for effective wound healing.

Biodegradability is a crucial feature for bioinks used in wound healing to ensure that they degrade at a rate that matches tissue regeneration, with some studies specifying rapid degradation rates, while others highlighted slow and controlled degradation to provide sustained therapeutic effects. Fast biodegradation can be beneficial for acute wounds, as it allows for rapid replacement of the scaffold with native tissue. However, in the context of chronic wounds, slow biodegradation is often more beneficial because it provides prolonged structural support, promoting stable and gradual tissue regeneration [61]. Ullah et al. (2023) [55] reported 85% weight loss, indicating significant biodegradability. Xia et al. (2022) [56] confirmed biodegradability, while Masri et al. (2024) [58] reported a slow degradation rate of 0.02 ± 0.005 mg/h. Bajuri et al. (2023) [54] noted the biodegradable scaffold properties of their autologous adipose tissue and fibrin gel bioink, though specific rates were not provided. 

The comprehensive analysis of bioink composition and characteristics in these studies emphasizes the importance of selecting appropriate cell types, densities, and preparation methods to optimize the therapeutic potential of 3D bioprinted constructs for chronic wound healing. The variability in bioink properties and their targeted applications underscores the need for continued research to refine and standardize bioink formulations to enhance clinical outcomes. These findings contribute to the growing body of knowledge supporting the use of 3D bioprinting technology in regenerative medicine, particularly in the treatment of chronic wounds.

### 3.5. Applications and Effectiveness of 3D Bioprinted Bioinks

The reviewed studies highlight various applications of 3D-printed bioinks in chronic wound healing, showcasing the innovation and effectiveness of different bioink formulations. Each study explores different bioink compositions, objectives, and biological functionalities, showcasing significant advancements in the field. Key applications include the delivery of cells, the creation of scaffold architectures tailored for wound healing, and the incorporation of bioactive compounds to enhance tissue regeneration. Table 5 provides a detailed summary of the objectives, print fidelity and stability, biological functionality, and the additional findings of each study. 

Schmitt et al. (2021) [52] were the first to apply 3D bioprinting with microfat tissue and collagen as a base bioink, maintaining print fidelity and supporting wound healing through cytokine release. Manso et al. (2023) [53] utilized xenogeneic MSCs to promote an anti-inflammatory environment, showing potential for restoring mast cells in diabetic skin. Bajuri et al. (2023) [54] highlighted the use of 3D-AMHAT with fibrin gel for diabetic foot ulcers, achieving significant wound size reduction and high-quality skin reconstruction without adverse events. Ullah et al. (2023) [55] focused on skin regeneration using PEO-CS-PMMA bioink with nicotinamide, demonstrating enhanced skin regeneration and effective DNA interaction. Xia et al. (2022) [56] were the first to incorporate curcumin into a GelMA scaffold for diabetic wound healing, resulting in reduced ROS, decreased apoptosis, and improved cell survival. Ma et al. (2021) [57] combined strontium silicate with bioink for vascularized skin regeneration, achieving enhanced cell proliferation and upregulation of angiogenic genes. Masri et al. (2023) [59] and (2024) [58] conducted detailed evaluations of hybrid bioinks, demonstrating improved wound healing, antioxidant properties, and enhanced cell migration and proliferation. Albanna et al. (2019) [60] used a mobile bioprinter with integrated imaging technology to create bilayered skin constructs directly on wounds. These innovations demonstrate ongoing efforts to enhance the therapeutic potential of bioprinted constructs for wound healing.

All reviewed studies reported high print fidelity and stability of their bioprinted constructs, which is crucial for maintaining the structural integrity and functionality of bioinks over time. This consistent print quality ensures that bioprinted tissues can effectively support the healing process. The biological functionality of the bioinks was a key focus across the studies. Many bioinks were designed to promote wound healing through various mechanisms. For instance, Manso et al., 2023 [53], demonstrated that their MSCs-laden alginate hydrogel promoted M2 macrophage polarization and supported an anti-inflammatory environment. Similarly, Schmitt et al. (2021) [52] reported the release of proinflammatory cytokines and the temporal release of IL-6, IL-8, and FGF-2, supporting wound healing. Ma et al., 2021 [57] showed that HDF-HUVEC- laden GAM-SS stimulated the VEGF, VE-cad, HIF-1α, eNOS-1 angiogenic gene expression and support blood vessel formation. Bajuri et al., 2023 [54], showed that their 3D-AMHAT with fibrin gel promoted high-quality skin reconstruction in diabetic foot ulcers (DFUs). Xia et al. (2022) [56] demonstrated reduced ROS, decreased apoptosis, improved ADSC survival, and enhanced wound healing with curcumin in GelMA scaffolds. Albanna et al. (2019) [60] demonstrated rapid wound closure, formation of normal skin, and reduced contraction, with early formation of defined epidermis and mature dermis layers in bioprinted wounds compared to cell-sprayed wounds.

Additional findings from these studies provide further insights into the broader potential and specific benefits of bioprinted bioinks. Schmitt et al., 2021 [52], noted the potential for weekly application of custom-designed autologous microfat grafts with bandage changes, while Manso et al. (2023) [53] highlighted the potential for restoring mast cells in diabetic skin. Ullah et al., 2023 [55], highlighted effective DNA interaction as an additional benefit of their PEO-CS-PMMA bioink to facilitate gene expression and cellular activities essential for wound healing. This includes promoting cell proliferation, differentiation, and migration, which are critical for tissue regeneration. Xia et al. (2022) [56] reported that curcumin significantly downregulated AGER and inhibited ROS production and apoptosis in ADSCs. Other studies, such as Masri et al. (2024) [58] and (2023) [59], noted high antioxidant capacity, improved rheological properties, optimal WVTR, slow biodegradation rate, and suitability for wound healing. Albanna et al. (2019) [60] emphasized the early formation of defined epidermis and mature dermis layers, demonstrating the superiority of bioprinted wounds over cell-sprayed wounds.

In summary, the applications and effectiveness of 3D-printed bioinks underscore the significant advancements being made in this field and the potential of this technology in regenerative medicine. By maintaining high print fidelity, promoting biological functionality, and providing additional benefits reported across these studies, it highlighted the potential of these bioinks to enhance chronic wound healing outcomes. Continued research and development in this area are essential to further optimize bioink formulations and maximize their therapeutic efficacy for clinical applications.

### 3.6. Summary of In Vivo Model Characteristics

The studies included in this review utilized various in vivo models to evaluate the effectiveness of 3D-printed bioinks for wound healing. Table 6 provides a detailed summary of the characteristics of these models, including model species, sex, age, defect area, defect size, time of sacrifice, health status, and post-operative care.

The predominant use of murine models, specifically C57BL/6 and Nu/nu athymic nude mice, allowed for a controlled environment to assess wound healing. The in vivo models used include C57BL/6 mice (Manso et al., 2023 [53]; Ma et al., 2021) [57], Nu/nu athymic nude mice (Xia et al., 2022 [56]; Albanna et al., 2019) [60], and Specific Pathogen Free (SPF) Yorkshire pigs (Albanna et al., 2019) [60]. These models were chosen for their relevance in mimicking human wound-healing processes. Additionally, Bajuri et al. (2023) [54] included human subjects, providing direct clinical relevance to their findings and bridging the gap between preclinical and clinical research. This range of models, from small rodents to large animal models and human subjects, highlights the translational potential of these findings. 

The age of the animal models ranged from 6 to 8 weeks, representing young adult mice, which are typically preferred for their robust health and consistent physiological responses. The sex of the animals was specified in most studies, with 24–36 male mice being predominantly used. Albanna et al. (2019) [60] did not specify the age but used six female Yorkshire pigs. For the human subjects in Bajuri et al. (2023) [54], the average age was 48.7 ± 14.9 years, including seven male and three female patients, reflecting a realistic demographic for chronic wound patients.

The defect areas were primarily located on the dorsal surface or back skin of the mice, with defect sizes varying across studies. Manso et al. (2023) [53] created defects measuring 1 cm^2^ on the dorsal surface, while Ma et al. (2021) [57] used circular full-thickness defects with a 1.5 cm^2^ diameter. Xia et al. (2022) [56] induced full skin defects measuring 1.5 cm^2^ on the dorsal surface of Nu/nu athymic nude mice. Albanna et al. (2019) [60] created 7.5 cm^2^ defects on the dorsal surface of mice and 100 cm^2^ defects on pigs. This range of defect sizes reflects the versatility of 3D-printed bioinks for treating various wound sizes. Bajuri et al. (2023) [54] studied foot ulcers in human patients, with an average defect size of 27.00 ± 43.99 cm^2^, highlighting the variability and complexity of real-world wound healing scenarios. 

The time of sacrifice post-surgery varies across studies, providing different durations for observing wound healing. The sacrifice time for the mice models varied from 14 to 21 days post-operation, allowing for the evaluation of short-term healing responses. Manso et al. (2023) [53] and Ma et al. (2021) [57] sacrificed their models at 15 days, whereas Xia et al. (2022) [56] conducted evaluations at both 14 and 21 days. Albanna et al. (2019) [60] sacrificed mice after 6 weeks and pigs after 8 weeks. In the Bajuri et al. (2023) [54] study involving human patients, the follow-up period was 12 weeks, providing a more extended observation period for chronic wound healing outcomes.

The health status of the model is critical for understanding the context of wound healing. Manso et al. (2023) [53] and Ma et al. (2021) [57] used diabetic (STZ-induced) C57BL/6 mice. Xia et al. (2022) [56] also used diabetic (STZ-induced) Nu/nu athymic nude mice. Albanna et al. (2019) [60] used Specific Pathogen Free (SPF) in Yorkshire pigs and female outbred athymic nude mice. Bajuri et al. (2023) [54] studied patients with Type II diabetes mellitus. The inclusion of diabetic models is particularly relevant for studying chronic wound healing. 

Post-operative care is controlled to ensure optimal recovery and accurate results. Manso et al. (2023) [53] maintained mice in a pathogen-free condition with a 12-h light/dark cycle at around 22 °C. Xia et al. (2022) [56] covered wounds with Vaseline gauze and a transparent film, which was changed every 2 days. Ma et al. (2021) [57] covered wounds with a scaffold, sterile gauze, and medical transparent dressing. Albanna et al. (2019) [60] used bandaging with sterile gauze and surgical tape, changing dressings every 3 days under anesthesia for both mice and pigs. Bajuri et al. (2023) [54] provided 12 weeks of follow-up for patients, noting no keloid formation, scarring, tissue contracture, or infection, indicating the safety and efficacy of the bioprinted constructs in a clinical setting. These detailed post-operative care protocols are crucial for supporting wound healing and ensuring the reliability of the results.

## 4. Discussion

Overview of 3D Bioprinting for Wound Healing

In recent years, the rapid evolution of 3D bioprinting technology for chronic wound healing has led to a significant diversity in bioprinting techniques, bioink formulations, and cell sources. This diversity has resulted in substantial variation in the techniques employed to evaluate printed constructs, making it challenging to compare outcomes across the literature. This scoping review aimed to thoroughly investigate the scope and nature of the evidence on specific practices associated with 3D printed bioinks for chronic wound healing in a structured manner.

The nine studies included in this review span multiple countries, including the USA, Brazil, Malaysia, Pakistan, and China, reflecting the global interest in and advancements in 3D bioprinting for wound healing. Most studies utilized extrusion-based bioprinting techniques due to their precision and control, although Albanna et al. (2019) [60] employed an innovative mobile in situ skin inkjet-based bioprinting method. The bioink compositions varied widely, encompassing natural polymers like methacrylated collagen (CMA), alginate hydrogel, fibrin gel, and gelatin methacryloyl (GelMA), as well as synthetic and hybrid polymers like PEO-CS-PMMA and gelatin-PVA. The use of these polymers in bioink formulations has proven beneficial due to their biocompatibility and ability to mimic the ECM [62,63]. This diversity underscores the ongoing exploration of optimizing bioink properties for specific wound types, such as chronic wounds, diabetic ulcers, and skin tissue regeneration.

Characteristics of 3D Bioprinting Techniques

The bioprinting techniques utilized across the studies demonstrated high print fidelity and stability, which are crucial for maintaining the structural integrity of the printed constructs [64]. The nozzle sizes and printing speeds were optimized for each bioink to ensure precise deposition, effective crosslinking, and prevent cell damage [65]. For instance, Schmitt et al. (2021) [52] used a 0.965 mm nozzle at 5 mm/s, while Xia et al. (2022) [56] employed a 0.21 mm nozzle at the same speed, highlighting the customization required for different bioinks [22]. Crosslinking methods, such as UV crosslinking, thermal initiation, and chemical crosslinking, were tailored to stabilize the printed structures. The printing environment, which was controlled in terms of temperature and sterility, further ensured the viability and functionality of the bioprinted constructs [66,67].

The reviewed studies emphasized the importance of optimizing printing parameters to achieve high print fidelity and stability. Schmitt et al. (2021) [52] and Manso et al. (2023) [53] reported stable print fidelity in their wound environments, while Bajuri et al. (2023) [54] demonstrated high print fidelity and stability in the treatment of diabetic foot ulcers (DFUs). These findings align with previous research indicating that extrusion-based bioprinting is suitable for chronic wound applications due to its ability to handle high-viscosity bioinks and maintain cell viability [68].

Bioink Composition and Characteristics

The bioinks reviewed demonstrated a range of properties, including high cell viability, appropriate mechanical strength, and biodegradability, which are all critical for effective wound healing. For example, Ullah et al. (2023) [55] reported a cell viability of 92% with their PEO-CS-PMMA bioink, while Xia et al. (2022) [56] achieved 90% cell viability using GelMA with curcumin, which is above the minimum threshold recovery of 85% for an ideal bioink, as suggested by [69]. Bioink preparation methods, such as mixing and free radical copolymerization, were designed to maintain the integrity and functionality of the incorporated cells. Mechanical properties such as viscosity and porosity were optimized to support cell adhesion, proliferation, and migration [68]. Biodegradability was also a key consideration, with bioinks like those used by Masri et al. (2023) [59] designed to degrade slowly, matching the pace of tissue regeneration. It is crucial to control the rate of degradation to ensure optimal healing before complete degradation, which can help prevent secondary infections. As mentioned by Shie et al., 2020 [70], even though the mechanical strength of hydrogels may decrease over time due to enzymatic digestion, an appropriate degree of degradation in GelMa allows encapsulated cells to remodel the microenvironment by secreting extracellular matrix (ECM) and associated proteins [70].

Several studies, including Schmitt et al. (2021) [52] and Ma et al. (2021) [57], have highlighted the significance of incorporating bioactive compounds within bioinks to enhance their therapeutic potential. Schmitt et al. (2021) [52] demonstrated sustained cytokine release and high cell viability using methacrylated collagen (CMA) and microfat, aligning with similar approaches in tissue engineering [71]. Ma et al. (2021) [57] utilized strontium silicate in their bioink to enhance vascularization. This is supported by previous studies that have emphasized the role of bioactive ceramics in boosting angiogenesis and tissue integration. For example, a study using fibroblast cell-laden collagen/strontium-doped calcium silicate (SrCS) bilayer scaffolds showed the potential to promote angiogenesis after 7 days [72]. The incorporation of bioactive compounds, such as curcumin in Xia et al. (2022) [56], aligns with the literature suggesting that bioinks containing bioactive molecules can significantly enhance therapeutic outcomes by reducing oxidative stress and promoting cell survival. For example, Ding et al. (2023) [73] demonstrated that bioactive printed hydrogel scaffolds incorporating molybdenum disulfide (MoS2) exhibit enhanced ROS-scavenging capability and impressive antibacterial properties, thereby facilitating the healing of infected chronic wounds [73].

Applications and Effectiveness

The applications of 3D-printed bioinks varied from promoting wound healing and reducing inflammation to enhancing skin regeneration and vascularization. Schmitt et al. (2021) [52] demonstrated the potential for weekly application of custom-designed autologous microfat grafts, while Manso et al. (2023) [53] highlighted the use of xenogeneic MSCs to promote an anti-inflammatory environment and restore mast cells in diabetic skin. Bajuri et al. (2023) [54] achieved significant wound size reduction and high-quality skin reconstruction in diabetic foot ulcers. Ullah et al. (2023) [55] and Xia et al. (2022) [56] reported enhanced skin regeneration and improved ADSC survival, respectively, demonstrating the effectiveness of their bioinks in promoting wound healing and cellular functionality. Ma et al. (2021) [57] showed increased expression of angiogenic genes and blood vessel formation, essential for vascularized skin regeneration. Masri et al. (2024 [58], 2023 [59]) reported a high antioxidant capacity and improved rheological properties, indicating the potential for long-term wound healing applications. Albanna et al. (2019) [60] demonstrated rapid wound closure and the formation of mature skin layers, showcasing the advanced capabilities of mobile bioprinting technology. These findings align with the broader literature on 3D bioprinting and chronic wound management [48,74,75]. Previous studies have similarly reported the benefits of using cell-laden bioinks for tissue regeneration and wound healing [23,76].

In Vivo Model Characteristics

The in vivo models used across the studies provided critical insights into the translational potential of printed bioinks. The models included diabetic mice and pigs, as well as human patients, reflecting a broad spectrum of wound-healing scenarios. The defect areas and sizes were carefully controlled to mimic clinical conditions, and the post-operative care protocols were designed to support wound healing and ensure the reliability of the results [77,78]. For example, Manso et al. (2023) [53] maintained mice in a pathogen-free environment, while Bajuri et al. (2023) [54] provided follow-up care for patients, noting no adverse events. The varying times of sacrifice allowed for both short-term and long-term observations, providing comprehensive data on the effectiveness of the printed bioinks. These in vivo models are crucial for evaluating the real-world applicability of 3D bioprinted constructs. Previous research has shown that animal models are essential for preclinical testing of tissue-engineered products, providing valuable information on biocompatibility, functionality, and long-term stability [79].

Clinical and Translational Potential

The clinical and translational potential of 3D-printed bioinks is vast. The ability to customize bioink formulations and bioprinting parameters allows the development of tailored treatments for individual patients. The high cell viability and functionality maintained in the bioprinted scaffold indicate a promising translation for their use in clinical settings to enhance wound healing processes [80]. For instance, the use of autologous cells in bioprinted constructs can minimize the risk of immune rejection and improve treatment outcomes [81]. Bajuri et al. (2023) [54] demonstrated the clinical application of autologous adipose tissue and fibrin gel in diabetic foot ulcers, achieving 70% complete healing within 12 weeks without adverse events. The translation of these technologies into clinical practice requires rigorous validation in clinical trials. Research on the cost-effectiveness and scalability of 3D bioprinting technologies is essential to ensure their widespread adoption in clinical settings. Policymakers should support initiatives that fund further research and development in this field, as the benefits of 3D-printed bioinks for chronic wound management are significant.

Strengths and Limitations

This review’s strength lies in its comprehensive approach, which adheres to established methodological frameworks and guidelines [49]. The inclusion criteria were rigorously applied to ensure the relevance of the selected studies. The inclusion of both preclinical and clinical studies provides a broad perspective on the translational potential of these technologies. A detailed analysis of bioink composition, 3D bioprinting techniques, and their applications offers valuable insights for future research and clinical practice.

However, there are limitations, including the small number of studies that met the inclusion criteria and the variability in study designs, bioink compositions, bioprinting parameters, and outcome measures across the reviewed studies, making direct comparisons challenging [74]. Some studies outsourced 3D bioprinting and did not provide detailed information on the printing techniques employed. Moreover, some studies lacked detailed methodology and information. These factors may limit the generalizability of our findings. Additionally, the exclusion of non-English articles may have led to the omission of relevant studies.

Limited long-term follow-up data in the included studies, particularly in clinical settings, restrict the ability to assess the durability and integration of bioprinted constructs over extended periods [82]. Extrusion-based bioprinting, while effective, generally encounters challenges related to cell viability due to the shear stress applied by the printing nozzle, cytotoxicity of crosslinkers, and incompatibility of hydrogel moduli with soft tissue cells [65]. Addressing these challenges through advanced bioprinting techniques and optimized bioink formulations will be critical for the future success of this technology in clinical applications.

Future Research Directions

While the results are promising, future research should focus on standardizing bioink formulations and printing parameters to enhance reproducibility and efficacy. Comparative studies exploring different bioink compositions and crosslinking methods can provide deeper insights into optimizing 3D bioprinting processes [68]. Furthermore, clinical trials are necessary to validate preclinical findings and assess the long-term outcomes of using 3D bioprinted bioinks in chronic wound management [83]. Additionally, the development of standardized protocols for bioprinting and post-operative care will be crucial for translating these innovations into clinical practice. Research on the cost-effectiveness and scalability of these technologies will also be crucial for their widespread adoption in clinical settings [84,85,86]. Investigating combinations of multiple cell types and bioactive compounds within bioinks could further enhance their therapeutic potential.

Moreover, integrating advanced imaging and monitoring technologies into the bioprinting process, as demonstrated by Albanna et al. (2019) [60], could further enhance the precision and effectiveness of 3D bioprinted constructs. Collaboration between researchers, clinicians, and industry will be essential to address the remaining challenges and bring these promising technologies to the market. In clinical practice, the customization of bioink compositions and printing parameters can be leveraged to develop tailored treatment approaches for chronic wounds, potentially improving patient outcomes and quality of life.

## 5. Conclusions

This scoping review underscores the transformative potential of 3D bioprinted bioinks in chronic wound healing. The advancements in bioink compositions, 3D bioprinting techniques, and their applications demonstrate significant progress in this field. The integration of living cells within bioinks and the customization of bioprinting parameters are pivotal for enhancing tissue regeneration and wound closure. While promising, further research is essential to optimize these technologies and ensure their successful clinical translation. The continued development and refinement of 3D bioprinting techniques hold significant promise for improving chronic wound management and patient outcomes.

## Figures and Tables

**Table 2:** Summary of the Included Studies.

No.	Study	Country	Study Design	3D Bioprinting Technique	Bioink Components	Wound Type	Assay Conducted	Key Outcomes	Study Limitations
1	[52]	USA	In vitro	Extrusion	Methacrylated Collagen (CMA)	Chronic wounds	Alamar blue assay	Sustained cytokine release, High cell viability, maintained print fidelity and stability	Limited in vivo data
2	[53]	Brazil	In vivo	Extrusion	Alginate Hydrogel	Chronic diabetic ulcers	Bioluminescence assay	Accelerated wound healing, increased mast cells, improved regenerative profile	Short-term study, limited to mice
3	[54]	Malaysia	Single-arm pilot study	Extrusion	Fibrin gel	Diabetic foot ulcers	Not stated (Clinical study)	70% complete healing within 12 weeks, significant wound size reduction, no adverse events	Small sample size, lack of control group, short follow-up period
4	[55]	Pakistan	In vivo	Extrusion	polyethylene oxide-co-Chitosan-co-poly (methylmethacrylic acid) (PEO-CS-PMMA), Nicotinamide	Full-thickness skin defect	Not stated	High reproducibility, improved mechanical properties, increased cell viability	lack of detailed methodology and analysis of biological interactions
5	[56]	China	In vitro and in vivo	Extrusion	Gelatin methacryloyl (GelMA), Curcumin	Diabetic wounds	Live/dead assay Apoptosis assay Western blot Caspase-3 activity assay	Reduced ROS, decreased apoptosis; improved ADSC cell survival, accelerated wound healing	Limited in vivo studies, need for long-term analysis, lack of comparison with other materials
6	[57]	China	In vitro and in vivo	Extrusion	SS-GAM (Strontium Silicate-Gellan Gum-Sodium Alginate—Methyl Cellulose)	Acute and chronic wounds	Live/dead assay Proliferation assay	Blood vessel formation, graft-host integration, enhanced cell proliferation, wound repair in vivo	short-term study
7	[58]	Malaysia	In vitro	Extrusion	Gelatin, Polyvinyl alcohol (PVA), Genipin	Potential for Chronic wounds	Live/dead assay Cell proliferation assay 3D cell migration In vitro wound scratch assay Immunocytochemistry DPPH and ABTS assay	Improved printability, high cell viability, increased wound healing rates	Limited in vivo studies, need for comparison with other materials
8	[59]	Malaysia	In vitro	Extrusion	Gelatin, Polyvinyl alcohol (PVA), Genipin	Potential for Chronic wounds	Live/dead assay Proliferation assay Scratch wound assays	Improved rheological properties, high cell viability (>90%), enhanced wound healing, reduced inflammation, optimal WVTR, slow biodegradation rate	Limited in vivo studies
9	[60]	USA	In vivo	Mobile in situ skin inkjet-based bioprinting with integrated imaging technology	Fibrin gel: fibrinogen- collagen	Acute and chronic wounds	Not stated	Improved wound closure, re-epithelialization, reduced contraction, formation of healthy, mature skin	potential for variability in outcomes due to the manual aspects of the bioprinting process and the limited sample size

ASCs: Adipose Stromal Cells, EPCs: Endothelial Progenitor Cells, MSCs: Mesenchymal Stem Cells, HDFs: Human Dermal Fibroblasts, ADSCs: Adipose-Derived Stem Cells, HUVECs: Human Umbilical Vascular Endothelial Cells, MUVECs: Murine Umbilical Vein Endothelial Cells, CMA: Methacrylated Collagen, PEO-CS-PMMA: Polyethylene Oxide-Chitosan-Poly (Methylmethacrylic Acid), GelMA: Gelatin Methacryloyl, SS: Strontium Silicate, PVA: Polyvinyl Alcohol.

**Table 3:** Characteristics of the 3D Bioprinting Techniques.

Study	Cell-Laden Bioink	Type of Polymer	3D Bioprinter Model	3D Bioprinting Technique	Type of Construct	Nozzle Size (mm)	Printing Speed (mm/s)	Printing Pressure (kPa)	Crosslinking Method	Printing Environment	Printing Shape	Crosslink Time (Min)
[52]	Microfat-laden Methacrylated Collagen (CMA)	Natural	REGEMAT3D (Granada, Spain)	Extrusion	Collagen construct	0.965	5	Not Available	UV crosslinking	Room temperature	Cube	1
[53]	MSCs-Laden Alginate Hydrogel	Natural	Available by the startup In Situ Cell Therapy	Extrusion	Hydrogel	Not Available	Not Available	Not Available	Not Available	Not Available	Not Available	Not Available
[54]	Autologous adipose tissue and fibrin gel	Natural	Dr. INVIVO clean chamber 3D bioprinter (ROKIT Healthcare, Seoul, Republic of Korea)	Extrusion	Graft	Not Available	Not Available	Not Available	Fibrin gel crosslinking	Clean chamber	According to wound size	Not Available
[55]	Fibroblast-PEO-CS-PMMA	Synthetic	Not Available	Extrusion	Hydrogel	0.25	5–8	20–45	Thermal initiation and chemical crosslinking with carbohydrazide (CH)	37 °C	Skin-tissue constructs	600
[56]	ADSCs (Adipose-Derived Stem Cells)-Gelatin Methacryloyl (GelMA)	Natural	BioArchitect Pro (Regenovo, Hangzhou, China)	Extrusion	Hydrogel	0.21	5	200	Blue light	20 °C nozzle, 18 °C platform	Circular mesh structure	0.33–0.5
[57]	HDF-HUVEC-GAM-SS	Natural	BioScaffolder 3.2 (GeSiM, Radeberg, Germany)	Extrusion	Hydrogel	0.25	Not Available	230–280	Ionic crosslinking with 1.6% CaCl_2_	Room temperature	hexagonal prism, 8mm side length, 1.4mm thickness	60
[58]	HDF-Gelatin-PVA	Hybrid (Natural and Synthetic)	Biogens XI (3D Gens, Shah Alam, Malaysia)	Extrusion	Hydrogel	0.3	5–10	150–200	Chemical crosslinking with Genipin	23 ± 2 °C, Sterile	Layer-by-layer 3D structures, Grid-like patterns	3
[59]	HDF-Gelatin-PVA	Hybrid (Natural and Synthetic)	Biogens XI (3D Gens, Malaysia)	Extrusion	Hydrogel	0.3	4000	Not Available	Chemical crosslinking with Genipin	23 ± 2 °C, Sterile	Grid-like patterns, 2.5 cm × 0.3 mm	Not Available
[60]	Fibroblasts-Keratinocytes-Fibrin Gel	Fibrinogen, collagen Natural	Custom-designed mobile bioprinter	inkjet	Hydrogel	0.26	1130.09–9417.45	6.89	Chemical crosslinking with Thrombin-induced gelation	Sterile, mobile operating room setup	Tailored to individual wound topography	15

ASCs: Adipose Stromal Cells, EPCs: Endothelial Progenitor Cells, MSCs: Mesenchymal Stem Cells, HDFs: Human Dermal Fibroblasts, ADSCs: Adipose-Derived Stem Cells, HUVECs: Human Umbilical Vascular Endothelial Cells, MUVECs: Murine Umbilical Vein Endothelial Cells, CMA: Methacrylated Collagen, PEO-CS-PMMA: Polyethylene Oxide-Chitosan-Poly (Methylmethacrylic Acid), GelMA: Gelatin Methacryloyl, SS: Strontium Silicate, PVA: Polyvinyl Alcohol.

**Table 4:** Bioink Composition and Characteristics.

Study	Cell-Laden Bioink	Cell Type	Cell Density	Max Cell Viability (%)	Bioink Preparation Method	Bioink Properties (e.g., Viscosity, Stiffness)	Biodegradability
[52]	Microfat-laden methacrylated collagen (CMA)	Adipose stromal cells (ASCs), Endothelial progenitor cells (EPCs)	1 × 10^6^ cells/gram	maintained for up to 10 days	Mixing	Not specified	Not specified
[53]	MSCs- Laden Alginate Hydrogel	Mesenchymal stem cells (MSCs, derived from the human umbilical cord)	1 × 10^5^ cells/cm²	Not specified	Mixing	Not specified	Not specified
[54]	Autologous adipose tissue and fibrin gel	Adipose stem cells	Not specified	Not specified	Mixed autologous adipose tissue with fibrin gel	Not specified	Biodegradable scaffold
[55]	Fibroblast-PEO-CS-PMMA Nicotinamide	Human Dermal Fibroblasts (HDFs)	0.1 × 10^6^ cells/mL	92%	Free radical copolymerization	Viscosity: 500–550 Pa·s, Thermal stability	85% weight loss
[56]	Gelatin methacryloyl (GelMA), Curcumin	Adipose-derived stem cells (ADSCs)	5 × 10^5^ cells/mL	90%	Mixing ADSCs with GelMA and Cur, crosslinked with blue light	High porosity, suitable mechanical properties	Yes
[57]	Strontium silicate microcylinders, Alginate, Gelatin	human umbilical vascular endothelial cells (HUVECs), human dermal fibroblasts (HDFs), murine umbilical vein endothelial cells (MUVEC), BALB/3T3 fibroblast	1 × 10^4^ cells/mL	Not specified	Mixing and ionic crosslinking	High viscosity, cell adhesion, high cellular activity, excellent proliferation, good mechanical strength	Not specified
[58]	HDF-Gelatin,(PVA)	Human dermal fibroblasts (HDFs)	1.5 × 10^6^ cells/mL	>90%	Mixed cells with hydrogel, chemical crosslinking with genipin	Appropriate mechanical properties, high porosity	0.02 ± 0.005 mg/h
[59]	HDF-Gelatin-PVA	Human dermal fibroblasts (HDFs)	1.5 × 10^6^ cells/mL	>90%	Mixed cells with hydrogel, chemical crosslinking with genipin	High viscosity, optimal WVTR, slow biodegradation rate, excellent interconnected porosity, achieve optimum printability	Slow, 0.018 ± 0.08 mg/h
[60]	Fibroblasts-Keratinocytes- Fibrin Gel	Dermal fibroblasts, epidermal keratinocytes	3.75 × 10^6^ cells/mL fibroblasts, 7.5 × 10^6^ cells/mL keratinocytes	High viability	Fibrinogen/collagen solution with cells printed directly onto the wound, followed by thrombin printing to form a gel	Suitable support for cellular viability, rapid crosslinking	Not specified

ASCs: Adipose Stromal Cells, EPCs: Endothelial Progenitor Cells, MSCs: Mesenchymal Stem Cells, HDFs: Human Dermal Fibroblasts, ADSCs: Adipose-Derived Stem Cells, HUVECs: Human Umbilical Vascular Endothelial Cells, MUVECs: Murine Umbilical Vein Endothelial Cells, CMA: Methacrylated Collagen, PEO-CS-PMMA: Polyethylene Oxide-Chitosan-Poly (Methylmethacrylic Acid), GelMA: Gelatin Methacryloyl, SS: Strontium Silicate, PVA: Polyvinyl Alcohol.

**Table 5:** Applications and Effectiveness of 3D Bioprinted Bioinks.

Study	Objective/Novelty	Print Fidelity and Stability	Biological Functionality	Additional Findings
[52]	First application of 3D bioprinting using microfat tissue with a collagen-based bioink	Maintained over time	Release proinflammatory cytokines, Temporal release of IL-6, IL-8, FGF-2, supports wound healing	Potential for weekly application of custom-designed autologous microfat grafts with bandage changes
[53]	Use of xenogeneic MSCs in biocuratives	Stable in the wound environment	Promotes M2 macrophage polarization, supports wound healing, promotes an anti-inflammatory environment (Increased TGF-β, IL-33, M2 markers)	Potential for restoring mast cells in diabetic skin
[54]	Use of 3D-AMHAT with fibrin gel for diabetic foot ulcers (DFUs)	High print fidelity and stability	Promoted wound healing with high-quality skin reconstruction	Significant wound size reduction, no adverse events, enhanced quality of healed skin
[55]	Use of PEO-CS-PMMA bioink with nicotinamide for skin regeneration	High print fidelity and stability	Enhanced skin regeneration	Effective DNA interaction
[56]	First study to incorporate curcumin in a 3D printed GelMA scaffold for diabetic wound healing	High print fidelity and stability	Reduced ROS, decreased apoptosis, improved ADSC survival, improved wound healing	Curcumin significantly downregulated AGER and inhibited ROS production and apoptosis in ADSCs
[57]	Combination of strontium silicate (SS) with bioink for vascularized skin regeneration	High print fidelity and stability	Enhanced cell proliferation, increased expression of angiogenic genes (VEGF, VE-cad, HIF-1α, eNOS-1), blood vessel formation, graft-host integration, and skin reconstruction	Improved mechanical properties
[58]	Comparison of injectable vs. 3D bioprinted hydrogels	High print fidelity and stability	Enhanced wound healing, antioxidant properties, improved cell migration, Promoted cell proliferation and adhesion	High antioxidant capacity
[59]	Detailed evaluation of hybrid bioinks’ physicochemical properties	High print fidelity and stability	Enhanced wound healing, antioxidant properties, improved cell migration, Promoted cell proliferation	Improved rheological properties, High antioxidant capacity, optimal WVTR, slow biodegradation rate, suitable for wound healing
[60]	Use of a mobile bioprinter with integrated imaging technology to create bilayered skin constructs directly on wounds, potentially improving wound healing outcomes compared to traditional methods.	High-precision delivery to specific locations of the wound	Rapid wound closure, formation of normal skin, reduced contraction	Early formation of defined epidermis and mature dermis layers in bioprinted wounds compared to cell-sprayed wounds

ASCs: Adipose Stromal Cells, EPCs: Endothelial Progenitor Cells, MSCs: Mesenchymal Stem Cells, HDFs: Human Dermal Fibroblasts, ADSCs: Adipose-Derived Stem Cells, HUVECs: Human Umbilical Vascular Endothelial Cells, MUVECs: Murine Umbilical Vein Endothelial Cells, CMA: Methacrylated Collagen, PEO-CS-PMMA: Polyethylene Oxide-Chitosan-Poly (Methylmethacrylic Acid), GelMA: Gelatin Methacryloyl, SS: Strontium Silicate, PVA: Polyvinyl Alcohol.

**Table 6:** Summary of in vivo Model Characteristics.

Study	Model	Sex	Age	Defect Area	Defect Size (cm^2^)	Time of Sacrifice (days)	Health Status	Post-Operative Care
[53]	C57BL/6 mice	>24 Male	8 weeks	Dorsal surface, Back skin	1 cm^2^	15 Days	Diabetic (T1D) induced by STZ	The pathogen-free condition housing room was set to a 12 h light/dark cycle with a temperature of about 22 °C
[54]	patients	Seven male, three female	48.7 ± 14.9 year	Foot ulcers	27.00 ± 43.99 cm^2^	Not applicable	Type II diabetes mellitus	12 weeks of follow-up. No keloid formation, scarring, tissue contracture, or infection noted
[56]	Nu/nu athymic nude mice	30 mice, Not specified	6 weeks	Dorsal surface/Full skin defect	1.5 cm^2^	14 and 21 days	diabetic (STZ-induced)	Covered with Vaseline gauze and transparent film, changed every 2 days
[57]	C57BL/6 mice	25 Male	7–8 weeks	Dorsal surface	1.5 cm^2^ diameter, circular full thickness	15 days	Diabetic (T1D) induced by STZ	The wound site was covered by a scaffold and bound up by sterile gauze and medical transparent dressing
[60]	outbred athymic Nu/nu nude mice, Specific Pathogen Free (SPF) Yorkshire pigs	36 female mice, six pigs	Not specified	Dorsal surface	7.5 cm^2^ for murine model, 100 cm^2^ for porcine model	6 weeks for a murine model; 8 weeks for a porcine model	Specific Pathogen Free (SPF) Yorkshire pigs, female outbred athymic nude (Nu/nu) mice	Bandaging with sterile gauze and surgical tape, dressing changes every 3 days under anesthesia

## Data Availability

Data are contained within the article and Supplementary Materials.

## Supplementary Materials

The following supporting information can be downloaded at: https://www.mdpi.com/article/10.3390/polym16172456/s1, Figure S1: Schematic showing the scale, design, and components of the skin bioprinter. Table S1: Provides detailed eligibility reasons for each paper for all 157 paper using Rayyan software.
