# Supplementary material for: Application of 3D-Printed Bioinks in Chronic Wound Healing: A Scoping Review
Source: Polymers (Basel). 2024 Aug 29;16(17):2456. doi: 10.3390/polym16172456 (PMC11397625; doi:10.3390/polym16172456)
Supplement: Supplementary file 1 [file polymers-16-02456-s001.zip › polymers-3137000-supplementary.pdf]

Supplemental Table S1. provides detailed eligibility reasons for each paper for all 157 paper using Rayyan software.

title	year	journal	issn	authors	doi	notes
Wound Healing: From Passive to Smart Dressings.	2021	Advanced healthcare materials	2192-2659 2192-2640	Farahani, Mojtaba and Shafiee, Abbas	10.1002/adhm.202100477	RAYYAN-INCLUSION: {"Asmaa"=>"Excluded", "Syafira"=>"Excluded"} RAYYAN-LABELS: not chronic | RAYYAN-EXCLUSION-REASONS: wrong publication type
Innovative Treatment Strategies to Accelerate Wound Healing: Trajectory and Recent Advancements.	2022	Cells	2073-4409	Kolimi, Praveen and Narala, Sagar and Nyavanandi, Dinesh and Youssef, Ahmed Adel Ali and Dudhipala, Narendar	10.3390/cells11152439	RAYYAN-INCLUSION: {"Asmaa"=>"Excluded", "Syafira"=>"Excluded"} RAYYAN-EXCLUSION-REASONS: wrong publication type
Skin Wound Healing Process and New Emerging Technologies for Skin Wound Care and Regeneration.	2020	Pharmaceutics	1999-4923	Tottoli, Erika Maria and Dorati, Rossella and Genta, Ida and Chiesa, Enrica and Pisani, Silvia and Conti, Bice	10.3390/pharmaceutics12080735	RAYYAN-INCLUSION: {"Asmaa"=>"Excluded", "Syafira"=>"Excluded"} RAYYAN-EXCLUSION-REASONS: wrong publication type
Double-network hydrogel enhanced by SS31-loaded mesoporous polydopamine nanoparticles: Symphonic collaboration of near-infrared photothermal antibacterial effect and mitochondrial maintenance for full-thickness wound healing in diabetes mellitus.	2023	Bioactive materials	2452-199X	Deng, Qing-Song and Gao, Yuan and Rui, Bi-Yu and Li, Xu-Ran and Liu, Po-Lin and Han, Zi-Yin and Wei, Zhan-Ying and Zhang, Chang-Ru and Wang, Fei and Dawes, Helen and Zhu, Tong-He and Tao, Shi-Cong and Guo, Shang-Chun	10.1016/j.bioactmat.2023.04.004	RAYYAN-INCLUSION: {"Asmaa"=>"Excluded", "Syafira"=>"Excluded"} RAYYAN-LABELS: not 3D bioprint | USER-NOTES: {"Asmaa"=>["Does not focus on 3D bioprinting (the title and abstract suggest a focus on hydrogel and nanoparticles, not specifically on 3D bioprinting).", "full text screen:\nThe study does not involve the use of 3D bioprinting technologies.\nThe hydrogel does not contain living cells."]}
Innovations in Stem Cell Therapy for Diabetic Wound Healing.	2023	Advances in wound care	2162-1918 2162-1934	Ho, Jasmine and Yue, Dominic and Cheema, Umber and Hsia, Henry	10.1089/wound.2021.0104	RAYYAN-INCLUSION: {"Asmaa"=>"Excluded", "Syafira"=>"Excluded"} RAYYAN-EXCLUSION-

				C. and Dardik, Alan		REASONS: wrong publication type
3D bioprinting: opportunities for wound dressing development.	2023	Biomedical materials (Bristol, England)	1748-605X 1748-6041	Wang, Xiudan and Wang, Yanhua and Teng, Yanjiao and Shi, Jie and Yang, Xinran and Ding, Ziling and Guo, Xiaoqin and Hou, Shike and Lv, Qi	10.1088/1748-605X/ace228	RAYYAN-INCLUSION: {"Asmaa"=>"Excluded", "Syafira"=>"Excluded"} RAYYAN-EXCLUSION-REASONS: wrong publication type
Cell-based dressings: A journey through chronic wound management.	2022	Biomaterials advances	2772-9508	Heras, Kevin Las and Igartua, Manoli and Santos-Vizcaino, Edorta and Hernandez, Rosa Maria	10.1016/j.bioadv.2022.212738	RAYYAN-INCLUSION: {"Asmaa"=>"Excluded", "Syafira"=>"Excluded"} RAYYAN-EXCLUSION-REASONS: wrong publication type
Bioprinted Hydrogels for Fibrosis and Wound Healing: Treatment and Modeling.	2022	Gels (Basel, Switzerland)	2310-2861	Guo, Jason L. and Longaker, Michael T.	10.3390/gels9010019	RAYYAN-INCLUSION: {"Asmaa"=>"Excluded", "Syafira"=>"Excluded"} RAYYAN-EXCLUSION-REASONS: wrong publication type
3D-printed electrospun fibres for wound healing.	2023	Wound repair and regeneration : official publication of the Wound Healing Society [and] the European Tissue Repair Society	1524-475X 1067-1927	Ye, Xilin and Zhang, Enshuo and Huang, Yaqin and Tian, Feng and Xue, Jiajia	10.1111/wrr.13119	RAYYAN-INCLUSION: {"Asmaa"=>"Excluded", "Syafira"=>"Excluded"} RAYYAN-LABELS: no cell | USER-NOTES: {"Asmaa"=>["The study does not involve the use of living cells in the bioinks"]}
Healing of Chronic Wounds: An Update of Recent Developments and Future Possibilities.	2019	Tissue engineering. Part B, Reviews	1937-3376 1937-3368	Kathawala, Mustafa Hussain and Ng, Wei Long and Liu, Dan and Naing, May Win and Yeong, Wai Yee and Spiller, Kara L. and Van Dyke, Mark and Ng, Kee Woei	10.1089/ten.TEB.2019.0019	RAYYAN-INCLUSION: {"Asmaa"=>"Excluded", "Syafira"=>"Excluded"} RAYYAN-EXCLUSION-REASONS: wrong publication type
Investigation on repairing diabetic foot ulcer based on 3D bio-printing Gel/dECM/Qcs	2023	Tissue & cell	1532-3072 0040-8166	Zhong, Yiming and Ma, Hailin and Lu, Yueqi and Cao, Liuyuan and Cheng, Yuen	10.1016/j.tice.2023.102213	RAYYAN-INCLUSION: {"Asmaa"=>"Excluded", "Syafira"=>"Maybe"} | RAYYAN-LABELS: no cell | USER-NOTES: {"Asmaa"=>["all good, only the bioink not

composite scaffolds.				Yee and Tang, Xin and Sun, Huanwei and Song, Kedong		include cells", "cells are not encapsulated in the formulation "]}
Three-Dimensional Printed Cellulose for Wound Dressing Applications.	2023	3D printing and additive manufacturing	2329-7670 2329-7662	Fahma, Farah and Firmanda, Afrinal and Cabral, Jaydee and Pletzer, Daniel and Fisher, John and Mahadik, Bhushan and Arnata, I. Wayan and Sartika, Dewi and Wulandari, Anting	10.1089/3dp.2021.0327	RAYYAN-INCLUSION: {"Asmaa"=>"Excluded", "Syafira"=>"Excluded"} RAYYAN-EXCLUSION-REASONS: wrong publication type
A review of diabetic wound models-Novel insights into diabetic foot ulcer.	2021	Journal of tissue engineering and regenerative medicine	1932-7005 1932-6254	Phang, Shou Jin and Arumugam, Bavani and Kuppusamy, Umah Rani and Fauzi, Mh Busra and Looi, Mee Lee	10.1002/term.3246	RAYYAN-INCLUSION: {"Asmaa"=>"Excluded", "Syafira"=>"Excluded"} RAYYAN-EXCLUSION-REASONS: wrong publication type
3D-bioprinted peptide coupling patches for wound healing.	2022	Materials today. Bio	2590-0064	Guan, Gaopeng and Lv, Qizhuang and Liu, Shengyuan and Jiang, Zhenzhen and Zhou, Chunxia and Liao, Weifang	10.1016/j.mtbio.2021.100188	RAYYAN-INCLUSION: {"Asmaa"=>"Excluded", "Syafira"=>"Excluded"} RAYYAN-LABELS: no cell | USER-NOTES: {"Asmaa"=>["Does not mention the use of bioinks with living cells (focuses on peptide coupling, which might not involve live cell bioprinting)."], "Syafira"=>["This study included peptide as bioink but without cells."]}
Emerging treatment strategies in wound care.	2022	International wound journal	1742-481X 1742-4801	Mirhaj, Marjan and Labbaf, Sheyda and Tavakoli, Mohamadreza and Seifalian, Alexander Marcus	10.1111/iwj.13786	RAYYAN-INCLUSION: {"Asmaa"=>"Excluded", "Syafira"=>"Excluded"} RAYYAN-EXCLUSION-REASONS: wrong publication type
Preclinical study of diabetic foot ulcers: From	2023	International wound journal	1742-481X	Du, Yuqing and Wang, Jie and Fan,	10.1111/iwj.14311	RAYYAN-INCLUSION: {"Asmaa"=>"Excluded", "Syafira"=>"Excluded"}

pathogenesis to vivo/vitro models and clinical therapeutic transformation.			1742-4801	Weijing and Huang, Renyan and Wang, Hongfei and Liu, Guobin		RAYYAN-EXCLUSION-REASONS: wrong publication type
Harnessing Multifaceted Next-Generation Technologies for Improved Skin Wound Healing.	2021	ACS applied bio materials	2576-6422	Bhar, Bibrita and Chouhan, Dimple and Pai, Nakhul and Mandal, Biman B.	10.1021/acsabm.1c00880	RAYYAN-INCLUSION: {"Asmaa"=>"Excluded", "Syafira"=>"Excluded"} RAYYAN-EXCLUSION-REASONS: wrong publication type
Methodologies of Autologous Skin Cell Spray Graft.	2022	Cureus	2168-8184	Shree, Anand and Vagga, Anjali A.	10.7759/cureus.31353	RAYYAN-INCLUSION: {"Asmaa"=>"Excluded", "Syafira"=>"Excluded"} RAYYAN-EXCLUSION-REASONS: wrong publication type
A Feasibility Study on 3D Bioprinting of Microfat Constructs Towards Wound Healing Applications.	2021	Frontiers in bioengineering and biotechnology	2296-4185	Schmitt, Trevor and Katz, Nathan and Kishore, Vipuil	10.3389/fbioe.2021.707098	RAYYAN-INCLUSION: {"Asmaa"=>"Included", "Syafira"=>"Included"}
The applications of 3D printing in wound healing: The external delivery of stem cells and antibiosis.	2023	Advanced drug delivery reviews	1872-8294 0169-409X	Yu, Qingtong and Wang, Qilong and Zhang, Linzhi and Deng, Wenwen and Cao, Xia and Wang, Zhe and Sun, Xuan and Yu, Jiangnan and Xu, Ximing	10.1016/j.addr.2023.114823	RAYYAN-INCLUSION: {"Asmaa"=>"Excluded", "Syafira"=>"Excluded"} RAYYAN-EXCLUSION-REASONS: wrong publication type
Planar-/Curvilinear-Bioprinted Tri-Cell-Laden Hydrogel for Healing Irregular Chronic Wounds.	2022	Advanced healthcare materials	2192-2659 2192-2640	Wu, Shin-Da and Dai, Niann-Tzyy and Liao, Chao-Yaug and Kang, Lan-Ya and Tseng, Yu-Wen and Hsu, Shan-Hui	10.1002/adhm.202201021	RAYYAN-INCLUSION: {"Asmaa"=>"Excluded", "Syafira"=>"Included"} | RAYYAN-LABELS: no access | USER-NOTES: {"Asmaa"=>["no access to this paper "]}
Advances in spray products for skin regeneration.	2022	Bioactive materials	2452-199X	Pleguezuelos-BeltrÃ¡n, Paula and GÃ¡lvez-MartÃn, Patricia and Nieto-GarcÃ-a, Daniel and Marchal, Juan Antonio	10.1016/j.bioactmat.2022.02.023	RAYYAN-INCLUSION: {"Asmaa"=>"Excluded", "Syafira"=>"Excluded"} RAYYAN-EXCLUSION-REASONS: wrong publication type

				and LÃ³pez-Ruiz, Elena		
3D printed wound constructs for skin tissue engineering: A systematic review in experimental animal models.	2023	Journal of biomedical materials research. Part B, Applied biomaterials	1552-4981 1552-4973	de Souza, Amanda and Martignago, Cintia Cristina Santi and Santo, Giovanna do Espirito and Sousa, Karolyne Dos Santos Jorge and Cruz, Matheus Almeida and Amaral, Gustavo Oliva and Parisi, Julia Risso and Estadella, DÃ©bora and Ribeiro, Daniel Araki and Granito, Renata Neves and Renno, Ana Claudia Muniz	10.1002/jbm.b.35237	RAYYAN-INCLUSION: {"Asmaa"=>"Excluded", "Syafira"=>"Excluded"} RAYYAN-EXCLUSION-REASONS: wrong publication type
Application of 3D Bioprinting Technologies to the Management and Treatment of Diabetic Foot Ulcers.	2020	Biomedicines	2227-9059	Tan, Chew Teng and Liang, Kun and Ngo, Zong Heng and Dube, Christabel Thembela and Lim, Chin Yan	10.3390/biomedicines8100441	RAYYAN-INCLUSION: {"Asmaa"=>"Excluded", "Syafira"=>"Excluded"} RAYYAN-EXCLUSION-REASONS: wrong publication type
Chronic wounds: Current status, available strategies and emerging therapeutic solutions.	2020	Journal of controlled release : official journal of the Controlled Release Society	1873-4995 0168-3659	Las Heras, Kevin and Igartua, Manoli and Santos-Vizcaino, Edorta and Hernandez, Rosa Maria	10.1016/j.jconrel.2020.09.039	RAYYAN-INCLUSION: {"Asmaa"=>"Excluded", "Syafira"=>"Excluded"} RAYYAN-EXCLUSION-REASONS: wrong publication type
Nanofiber Aerogels with Precision Macrochannels and LL-37-Mimic Peptides Synergistically Promote Diabetic Wound Healing.	2023	Advanced functional materials	1616-301X 1616-3028	John, Johnson V. and Sharma, Navatha Shree and Tang, Guosheng and Luo, Zeyu and Su, Yajuan and Weihs, Shelbie and Shahriar, S.	10.1002/adfm.202206936	RAYYAN-INCLUSION: {"Asmaa"=>"Excluded", "Syafira"=>"Excluded"} | USER-NOTES: {"Asmaa"=>["Does not mention the use of bioinks with living cells (focuses on nanofiber aerogels, not directly on 3D bioprinting with cellular bioinks)."], "Syafira"=>["Not focusing on 3D-

				M. Shatil and Wang, Guangshun and McCarthy, Alec and Dyke, Justin and Zhang, Yu Shrike and Khademhosseini, Ali and Xie, Jingwei		bioprinting and do not incorporate cell."]}
Recent Advances in the Design of Three-Dimensional and Bioprinted Scaffolds for Full-Thickness Wound Healing.	2022	Tissue engineering. Part B, Reviews	1937-3376 1937-3368	Tan, Shi Hua and Ngo, Zong Heng and Sci, Dip Biomed and Leavesley, David and Liang, Kun	10.1089/ten.TEB.2020.0339	RAYYAN-INCLUSION: {"Asmaa"=>"Excluded", "Syafira"=>"Excluded"} RAYYAN-EXCLUSION-REASONS: wrong publication type
Bioengineering Skin Substitutes for Wound Management-Perspectives and Challenges.	2024	International journal of molecular sciences	1422-0067	Kondej, Karolina and Zawrzykraj, MaÅ‚gorzata and Czerwiec, Katarzyna and DeptuÅ‚a, Milena and TymiÅ„ska, Agata and PikuÅ‚a, MichaÅ‚	10.3390/ijms25073702	RAYYAN-INCLUSION: {"Asmaa"=>"Excluded", "Syafira"=>"Excluded"} RAYYAN-EXCLUSION-REASONS: wrong publication type
Recent Developments in 3D-(Bio)printed Hydrogels as Wound Dressings.	2024	Gels (Basel, Switzerland)	2310-2861	Kammona, Olga and Tsanaktsidou, Evgenia and Kiparissides, Costas	10.3390/gels10020147	RAYYAN-INCLUSION: {"Asmaa"=>"Excluded", "Syafira"=>"Excluded"} RAYYAN-EXCLUSION-REASONS: wrong publication type
Biofabrication of thick vascularized neo-pedicle flaps for reconstructive surgery.	2019	Translational research : the journal of laboratory and clinical medicine	1878-1810 1931-5244	Stephens, Chelsea J. and Spector, Jason A. and Butcher, Jonathan T.	10.1016/j.trsl.2019.05.003	RAYYAN-INCLUSION: {"Asmaa"=>"Excluded", "Syafira"=>"Excluded"} RAYYAN-EXCLUSION-REASONS: wrong publication type
Biofabrication of Cellulose-based Hydrogels for Advanced Wound Healing: A Special Emphasis on 3D Bioprinting.	2023	Macromolecular bioscience	1616-5195 1616-5187	Tabatabaei Hosseini, Behina Sadat and Meadows, Kieran and Gabriel, Vincent and Hu, Jinguang and Kim, Keekyoung	10.1002/mabi.202300376	RAYYAN-INCLUSION: {"Asmaa"=>"Excluded", "Syafira"=>"Excluded"} RAYYAN-EXCLUSION-REASONS: wrong publication type
Extracellular Matrices as	2023	Pharmaceutics	1999-4923	Zhao, Peng and Yang,	10.3390/pharmaceutics15122771	RAYYAN-INCLUSION: {"Asmaa"=>"Excluded",

Bioactive Materials for In Situ Tissue Regeneration.				Fengbo and Jia, Xiaoli and Xiao, Yuqin and Hua, Chao and Xing, Malcolm and Lyu, Guozhong		"Syafira"=>"Excluded"} RAYYAN-EXCLUSION-REASONS: wrong publication type
Copper-Epigallocatechin Gallate Enhances Therapeutic Effects of 3D-Printed Dermal Scaffolds in Mitigating Diabetic Wound Scarring.	2023	ACS applied materials & interfaces	1944-8252 1944-8244	Hu, Yanke and Xiong, Yahui and Zhu, Yongkang and Zhou, Fei and Liu, Xiaogang and Chen, Shuying and Li, Zhanpeng and Qi, Shaohai and Chen, Lei	10.1021/acsami.3c04733	RAYYAN-INCLUSION: {"Asmaa"=>"Excluded", "Syafira"=>"Excluded"} | USER-NOTES: {"Asmaa"=>["no cell/bioink "], "Syafira"=>["This study focus on 3D printed instead of 3D bioprinted. This study do not mention about bioinks (Cell encapsulation)"]}
Leveraging the advancements in functional biomaterials and scaffold fabrication technologies for chronic wound healing applications.	2022	Materials horizons	2051-6355 2051-6347	Ali Zahid, Alap and Chakraborty, Aishik and Shamiya, Yasmeen and Ravi, Shruthi Polla and Paul, Arghya	10.1039/d2mh00115b	RAYYAN-INCLUSION: {"Asmaa"=>"Excluded", "Syafira"=>"Excluded"} RAYYAN-EXCLUSION-REASONS: wrong publication type
Combining microfluidics and coaxial 3D-bioprinting for the manufacturing of diabetic wound healing dressings.	2023	Biomaterials advances	2772-9508	Fratini, Costanza and Weaver, Edward and Moroni, Sofia and Irwin, Robyn and Dallal Bashi, Yahya H. and Uddin, Shahid and Casettari, Luca and Wylie, Matthew P. and Lamprou, Dimitrios A.	10.1016/j.bioadv.2023.213557	RAYYAN-INCLUSION: {"Asmaa"=>"Excluded", "Syafira"=>"Included", "Izzah Fadilah"=>"Excluded"} | RAYYAN-LABELS: no cell | USER-NOTES: {"Asmaa"=>["absence of encapsulated living cells in the bioink."]}
Bioinspired 3D-printed scaffold embedding DDAB-nano ZnO/nanofibrous microspheres for regenerative diabetic wound healing.	2023	Biofabrication	1758-5090 1758-5082	Metwally, Walaa M. and El-Habashy, Salma E. and El-Hosseiny, Lobna S. and Essawy, Marwa M. and Eltaher, Hoda M. and El-	10.1088/1758-5090/acfd60	RAYYAN-INCLUSION: {"Asmaa"=>"Excluded", "Syafira"=>"Excluded"} | USER-NOTES: {"Asmaa"=>["Does not focus on 3D bioprinting (although using 3D printing, the focus is on material properties rather than bioprinting with living cells)"],

				Khordagui, Labiba K.		"Syafira"=>["Not bioink (cell encapsulation)"]}
Collagen-Hyaluronic Acid Composite Hydrogels with Applications for Chronic Diabetic Wound Repair.	2023	ACS biomaterials science & engineering	2373-9878	Liang, Mujiao and Dong, Lina and Guo, Zhongwei and Liu, Liming and Fan, Zixin and Wei, Cunyue and Mi, Shengli and Sun, Wei	10.1021/acsbiomaterials.3c00695	RAYYAN-INCLUSION: {"Asmaa"=>"Excluded", "Syafira"=>"Excluded"} | USER-NOTES: {"Asmaa"=>["Does not mention the use of bioinks with living cells (focuses on composite hydrogels without clear mention of 3D bioprinting)."], "Syafira"=>["This study do not mention bioinks as their formulation. "]}
3D Printing of Strontium Silicate Microcylinder-Containing Multicellular Biomaterial Inks for Vascularized Skin Regeneration.	2021	Advanced healthcare materials	2192-2659 2192-2640	Ma, Jingge and Qin, Chen and Wu, Jinfu and Zhang, Hongjian and Zhuang, Hui and Zhang, Meng and Zhang, Zhaowenbin and Ma, Lingling and Wang, Xin and Ma, Bing and Chang, Jiang and Wu, Chengtie	10.1002/adhm.202100523	RAYYAN-INCLUSION: {"Asmaa"=>"Included", "Syafira"=>"Excluded", "Izzah Fadilah"=>"Excluded"} | USER-NOTES: {"Asmaa"=>["after full text screening: bioink contains living cells (HUVECs and HDFs).\nbioprinting of cell-laden SS-GAM scaffolds, where human dermal fibroblasts (HDFs) and human umbilical vascular endothelial cells (HUVECs) are incorporated into the bioink"]}
Recent advances in 3D printed cellulose-based wound dressings: A review on in vitro and in vivo achievements.	2023	Carbohydrate polymers	1879-1344 0144-8617	Pita-Vilar, Maria and Concheiro, Angel and Alvarez-Lorenzo, Carmen and Diaz-Gomez, Luis	10.1016/j.carbpol.2023.121298	RAYYAN-INCLUSION: {"Asmaa"=>"Excluded", "Syafira"=>"Excluded"} RAYYAN-EXCLUSION-REASONS: wrong publication type
Current Insight of Printability Quality Improvement Strategies in Natural-Based Bioinks for Skin Regeneration and Wound Healing.	2021	Polymers	2073-4360	Masri, Syafira and Fauzi, Mh Busra	10.3390/polym13071011	RAYYAN-INCLUSION: {"Asmaa"=>"Excluded", "Syafira"=>"Excluded"} RAYYAN-EXCLUSION-REASONS: wrong publication type
Cerium Oxide Nanoparticles (Nanoceria): Hopes in Soft Tissue Engineering.	2020	Molecules (Basel, Switzerland)	1420-3049	Sadidi, Hossein and Hooshmand, Sara and Ahmadabadi, Ali and Javad	10.3390/molecules25194559	RAYYAN-INCLUSION: {"Asmaa"=>"Excluded", "Syafira"=>"Excluded"} RAYYAN-INCLUSION:

				Hosseini, Seyed and Baino, Francesco and Vatanpour, Morvarid and Kargozar, Saeid		{"Asmaa"=>"Excluded", "Syafira"=>"Excluded"}
Cellular human tissue-engineered skin substitutes investigated for deep and difficult to heal injuries.	2021	NPJ Regenerative medicine	2057-3995	Sierra-SÃ¡nchez, Ã□lvaro and Kim, Kevin H. and Blasco-Morente, Gonzalo and Arias-Santiago, Salvador	10.1038/s41536-021-00144-0	RAYYAN-INCLUSION: {"Asmaa"=>"Excluded", "Syafira"=>"Excluded"} | RAYYAN-EXCLUSION-REASONS: wrong publication type
3D printed carboxymethyl cellulose scaffolds for autologous growth factors delivery in wound healing.	2022	Carbohydrate polymers	1879-1344 0144-8617	Diaz-Gomez, Luis and Gonzalez-Prada, Iago and Millan, Rosendo and Da Silva-Candal, Andres and Bugallo-Casal, Ana and Campos, Francisco and Concheiro, Angel and Alvarez-Lorenzo, Carmen	10.1016/j.carbpol.2021.118924	RAYYAN-INCLUSION: {"Asmaa"=>"Excluded", "Syafira"=>"Excluded"} | USER-NOTES: {"Asmaa"=>["Does not focus on 3D bioprinting or chronic wounds (focuses on drug delivery systems using 3D printed scaffolds but not specifically bioprinting with cellular bioinks)."]}
Cellular Interaction of Human Skin Cells towards Natural Bioink via 3D-Bioprinting Technologies for Chronic Wound: A Comprehensive Review.	2022	International journal of molecular sciences	1422-0067	Masri, Syafira and Zawani, Mazlan and Zulkiflee, Izzat and Salleh, Atiqah and Fadilah, Nur Izzah Md and Maarof, Manira and Wen, Adzim Poh Yuen and Duman, Fatih and Tabata, Yasuhiko and Aziz, Izhar Abd and Bt Hj Idrus, Ruszymah and Fauzi, Mh Busra	10.3390/ijms23010476	RAYYAN-INCLUSION: {"Asmaa"=>"Excluded", "Syafira"=>"Excluded"} RAYYAN-EXCLUSION-REASONS: wrong publication type

3D-Printed Hydrogel-Filled Microneedle Arrays.	2021	Advanced healthcare materials	2192-2659 2192-2640	Barnum, Lindsay and Quint, Jacob and Derakhshandeh, Hossein and Samandari, Mohamadmahdi and Aghabaglou, Fariba and Farzin, Ali and Abbasi, Laleh and Bencherif, Sidi and Memic, Adnan and Mostafalu, Pooria and Tamayol, Ali	10.1002/adhm.202001922	RAYYAN-INCLUSION: {"Asmaa"=>"Excluded", "Syafira"=>"Excluded"} | USER-NOTES: {"Asmaa"=>["hydrogel for drug delivery!"]}
Stem cell-derived small extracellular vesicles embedded into methacrylated hyaluronic acid wound dressings accelerate wound repair in a pressure model of diabetic ulcer.	2023	Journal of nanobiotechnology	1477-3155	Ferroni, Letizia and D'Amora, Ugo and Gardin, Chiara and Leo, Sara and Dalla Paola, Luca and Tremoli, Elena and Giuliani, Alessandro and CalzÃ , Laura and Ronca, Alfredo and Ambrosio, Luigi and Zavan, Barbara	10.1186/s12951-023-02202-9	RAYYAN-INCLUSION: {"Asmaa"=>"Excluded", "Syafira"=>"Included", "Izzah Fadilah"=>"Excluded"} | RAYYAN-LABELS: no cell | USER-NOTES: {"Asmaa"=>["It is EV but the is no cell included "]}
3D printed arrowroot starch-gellan scaffolds for wound healing applications.	2024	International journal of biological macromolecules	1879-0003 0141-8130	Joseph, Abey and Muhammad L, Fathah and S Vijayan, Athira and Xavier, Joseph and K B, Megha and Karthikeyan, Akash and Gopinath, Nigina and P V, Mohanan and Nair, Baiju G.	10.1016/j.ijbiomac.2024.130604	RAYYAN-INCLUSION: {"Asmaa"=>"Excluded", "Syafira"=>"Included", "Izzah Fadilah"=>"Included"} | RAYYAN-LABELS: not 3D bioprint

3D bioprinting of a gradient stiffened gelatin-alginate hydrogel with adipose-derived stem cells for full-thickness skin regeneration.	2023	Journal of materials chemistry. B	2050-7518 2050-750X	Ma, Yuan and Wang, Yilin and Chen, Danni and Su, Ting and Chang, Qiang and Huang, Wenhua and Lu, Feng	10.1039/d2tb02200a	RAYYAN-INCLUSION: {"Asmaa"=>"Excluded", "Syafira"=>"Maybe"} | RAYYAN-LABELS: no access | USER-NOTES: {"Asmaa"=>["3D bioprinted bioinks containing living adipose-derived stem cells for chronic wound healing and full-thickness skin regeneration"]}
Current Trends on Innovative Technologies in Topical Wound Care for Advanced Healing and Management.	2023	Current drug research reviews	2589-9783 2589-9775	Saifullah, Qazi and Sharma, Abhishek	10.2174/0125899775262048230925054922	RAYYAN-INCLUSION: {"Asmaa"=>"Excluded", "Syafira"=>"Excluded"} | RAYYAN-EXCLUSION-REASONS: wrong publication type
The efficacy of a paeoniflorin-sodium alginate-gelatin skin scaffold for the treatment of diabetic wound: An in vivo study in a rat model.	2022	Biomedicine & pharmacotherapy = Biomedecine & pharmacotherapie	1950-6007 0753-3322	Yu, Haiyang and Gong, Wen and Mei, Junhao and Qin, Lihao and Piao, Zeyu and You, Deshu and Gu, Wenxian and Jia, Zhongzhi	10.1016/j.biopha.2022.113165	RAYYAN-INCLUSION: {"Asmaa"=>"Excluded", "Syafira"=>"Included", "Izzah Fadilah"=>"Excluded"} | RAYYAN-LABELS: no cell | USER-NOTES: {"Asmaa"=>["Full Text Review: The full text confirms the use of paeoniflorin, sodium alginate, and gelatin to develop the bioink but does not mention the incorporation of living cells."]}
Future applications of 3D bioprinting: A promising technology for treating recessive dystrophic epidermolysis bullosa.	2022	Experimental dermatology	1600-0625 0906-6705	Popp, Courtney M. and Miller, William C. and Eide, Cindy R. and Tolar, Jakub	10.1111/exd.14484	RAYYAN-INCLUSION: {"Asmaa"=>"Excluded", "Syafira"=>"Excluded"} | RAYYAN-EXCLUSION-REASONS: wrong publication type
Antioxidant-enriched autologous biogel promoted diabetic wound healing by remodeling inherent posttraumatic inflammatory patterning and restoring compromised	2022	Regenerative biomaterials	2056-3418 2056-3426	Yang, Yixi and Wang, Le and Zhou, Yonglin and He, Yijun and Lin, Shaozhang and Zeng, Yuwei and Zhou, Yunhe and Li, Wei and He, Zaopeng and	10.1093/rb/rbac023	RAYYAN-INCLUSION: {"Asmaa"=>"Excluded", "Syafira"=>"Excluded"} | RAYYAN-LABELS: not 3D bioprint

microenvironment homeostasis.				Zhao, Qi and Chen, Lihao and Li, Zijie and Wang, Wenhao and Zhang, Zhi-Yong		
A 4D Printed Adhesive, Thermo-Contractile, and Degradable Hydrogel for Diabetic Wound Healing.	2024	Advanced healthcare materials	2192-2659 2192-2640	Lu, Zhe and Cui, Jingjing and Liu, Fukang and Liang, Chen and Feng, Shiwei and Sun, Yongding and Gao, Weizi and Guo, Yunlong and Zhang, Biao and Huang, Wei	10.1002/adhm.202303499	RAYYAN-INCLUSION: {"Asmaa"=>"Excluded", "Syafira"=>"Excluded"} | RAYYAN-LABELS: no cell | USER-NOTES: {"Asmaa"=>["4d "]}
A nanofibrous membrane loaded with doxycycline and printed with conductive hydrogel strips promotes diabetic wound healing in vivo.	2022	Acta biomaterialia	1878-7568 1742-7061	Cao, Wangbei and Peng, Shiqiao and Yao, Yuejun and Xie, Jieqi and Li, Shifen and Tu, Chenxi and Gao, Changyou	10.1016/j.actbio.2022.08.048	RAYYAN-INCLUSION: {"Asmaa"=>"Excluded", "Syafira"=>"Excluded"} | RAYYAN-LABELS: no cell | USER-NOTES: {"Asmaa"=>["Does not mention the use of bioinks with living cells (focuses on nanofibers, not explicitly on 3D bioprinting with cells)."]}
3D printable, injectable amyloid-based composite hydrogel of bovine serum albumin and aloe vera for rapid diabetic wound healing.	2023	Journal of materials chemistry. B	2050-7518 2050-750X	Naik, Kaustubh and Singh, Priyanka and Yadav, Monika and Srivastava, Saurabh Kr and Tripathi, Shikha and Ranjan, Rahul and Dhar, Prodyut and Verma, Anita Kamra and Chaudhary, Shilpi and Parmar, Avanish Singh	10.1039/d3tb01151h	RAYYAN-INCLUSION: {"Asmaa"=>"Excluded", "Syafira"=>"Included", "Izzah Fadilah"=>"Excluded"} | RAYYAN-LABELS: no cell | USER-NOTES: {"Asmaa"=>["does not encapsulating living cells within the hydrogel formulation for 3D printing"]}
Xenogeneic mesenchymal stem cell biocurative improves skin wounds healing in diabetic mice	2023	Regenerative therapy	2352-3204	Manso, Gabriel Martins da Costa and Elias-Oliveira, Jefferson and	10.1016/j.reth.2022.12.006	RAYYAN-INCLUSION: {"Asmaa"=>"Included", "Syafira"=>"Included"}

by increasing mast cells and the regenerative profile.				GuimarÃ£es, Jhefferson Barbosa and Pereira, Ã□talo Sousa and Rodrigues, Vanessa Fernandes and Burger, Beatriz and Fantacini, Daianne Maciely Carvalho and de Souza, Lucas Eduardo Botelho and Rodrigues, Hosana Gomes and Bonato, VÃ¢nia Luiza Deperon and Silva, JoÃ£o Santana and Ramos, Simone GusmÃ£o and Tostes, Rita Cassia and Manfiolli, Adriana Oliveira and Caliari-Oliveira, Carolina and Carlos, Daniela		
In vivo evaluation of an electrospun and 3D printed cellular delivery device for dermal wound healing.	2020	Journal of biomedical materials research. Part B, Applied biomaterials	1552-4981 1552-4973	Clohessy, Ryan M. and Cohen, David J. and Stumbraite, Karolina and Boyan, Barbara D. and Schwartz, Zvi	10.1002/jbm.b.34587	RAYYAN-INCLUSION: {"Asmaa"=>"Excluded", "Syafira"=>"Excluded"} | RAYYAN-LABELS: no cell | USER-NOTES: {"Asmaa"=>["device "]}
New Paradigm in Diabetic Foot Ulcer Grafting Techniques Using 3D-Bioprinted Autologous Minimally Manipulated Homologous	2023	Gels (Basel, Switzerland)	2310-2861	Bajuri, Mohd Yazid and Kim, Jeehee and Yu, Yeongseo and Shahul Hameed, Muhammad Shazwan	10.3390/gels9010066	RAYYAN-INCLUSION: {"Asmaa"=>"Included", "Syafira"=>"Included"}

Adipose Tissue (3D-AMHAT) with Fibrin Gel Acting as a Biodegradable Scaffold.						
A facile one-stone-two-birds strategy for fabricating multifunctional 3D nanofibrous scaffolds.	2023	Biomaterials science	2047-4849 2047-4830	Amarjargal, Altangerel and Moazzami Goudarzi, Zahra and Cegielska, Olga and Gradys, Arkadiusz and Kolbuk, Dorota and Kalaska, Bartlomiej and RuszczyÅ„ska, Anna and Sajkiewicz, Pawel	10.1039/d3bm00837a	RAYYAN-INCLUSION: {"Asmaa"=>"Excluded", "Syafira"=>"Excluded"} | RAYYAN-LABELS: not chronic ,fabrication only no biological
"Sandwich-like" structure electrostatic spun micro/nanofiber polylactic acid-polyvinyl alcohol-polylactic acid film dressing with metformin hydrochloride and puerarin for enhanced diabetic wound healing.	2023	International journal of biological macromolecules	1879-0003 0141-8130	Liao, Minjian and Jian, Xuewen and Zhao, Yanyan and Fu, Xuewei and Wan, Meiling and Zheng, Wenxu and Dong, Xianming and Zhou, Wuyi and Zhao, Hui	10.1016/j.ijbiomac.2023.127223	RAYYAN-INCLUSION: {"Asmaa"=>"Excluded", "Syafira"=>"Excluded"} | RAYYAN-LABELS: not 3D bioprint
Injectable Crosslinked Genipin Hybrid Gelatin-PVA Hydrogels for Future Use as Bioinks in Expediting Cutaneous Healing Capacity: Physicochemical Characterisation and Cytotoxicity Evaluation.	2022	Biomedicines	2227-9059	Masri, Syafira and Maarof, Manira and Mohd, Nor Fatimah and Hiraoka, Yosuke and Tabata, Yasuhiko and Fauzi, Mh Busra	10.3390/biomedicines10102651	RAYYAN-INCLUSION: {"Asmaa"=>"Excluded", "Syafira"=>"Included"} | RAYYAN-LABELS: no cell,not chronic
Biomimetic In Vitro Model of Cell Infiltration into Skin Scaffolds for Pre-Screening	2019	Cells	2073-4409	Ballesteros-Cillero, Rafael and Davison-Kotler, Evan and Kohli,	10.3390/cells8080917	RAYYAN-INCLUSION: {"Asmaa"=>"Excluded", "Syafira"=>"Excluded"} | RAYYAN-LABELS: no cell,not chronic |

and Testing of Biomaterial-Based Therapies.				Nupur and Marshall, William S. and GarcÃa-Gareta, Elena		USER-NOTES: {"Syafira"=>["cell seeding (culture on top)"]}
Multifunctionalised skin substitute of hybrid gelatin-polyvinyl alcohol bioinks for chronic wound: injectable vs. 3D bioprinting.	2024	Drug delivery and translational research	2190-3948 2190-393X	Masri, Syafira and Fadilah, Nur Izzah Md and Hao, Looi Qi and Maarof, Manira and Tabata, Yasuhiko and Hiraoka, Yosuke and Fauzi, Mh Busra	10.1007/s13346-023-01447-z	RAYYAN-INCLUSION: {"Asmaa"=>"Included", "Syafira"=>"Included"}
Development of highly-reproducible hydrogel based bioink for regeneration of skin-tissues via 3-D bioprinting technology.	2023	International journal of biological macromolecules	1879-0003 0141-8130	Ullah, Faheem and Javed, Fatima and Mushtaq, Irrum and Rahman, Latif-Ur and Ahmed, Nazir and Din, Israf Ud and Alotaibi, Mshari A. and Alharthi, Abdulrahman I. and Ahmad, Akil and Bakht, M. Afroz and Khan, Fayyaz and Tasleem, S.	10.1016/j.ijbiomac.2022.123131	RAYYAN-INCLUSION: {"Asmaa"=>"Included", "Syafira"=>"Included"}
Battery-free, wireless soft sensors for continuous multi-site measurements of pressure and temperature from patients at risk for pressure injuries.	2021	Nature communications	2041-1723	Oh, Yong Suk and Kim, Jae-Hwan and Xie, Zhaoqian and Cho, Seokjoo and Han, Hyeonseok and Jeon, Sung Woo and Park, Minsu and Namkoong, Myeong and Avila, Raudel and Song, Zhen and Lee, Sung-Uk and Ko, Kabseok and Lee, Jungyup and Lee, Je-Sang and	10.1038/s41467-021-25324-w	RAYYAN-INCLUSION: {"Asmaa"=>"Excluded", "Syafira"=>"Excluded"}

				Min, Weon Gi and Lee, Byeong-Ju and Choi, Myungwoo and Chung, Ha Uk and Kim, Jongwon and Han, Mengdi and Koo, Jahyun and Choi, Yeon Sik and Kwak, Sung Soo and Kim, Sung Bong and Kim, Jeonghyun and Choi, Jungil and Kang, Chang-Mo and Kim, Jong Uk and Kwon, Kyeongha and Won, Sang Min and Baek, Janice Mihyun and Lee, Yujin and Kim, So Young and Lu, Wei and Vazquez-Guardado, Abraham and Jeong, Hyoyoung and Ryu, Hanjun and Lee, Geumbee and Kim, Kyuyoung and Kim, Seunghwan and Kim, Min Seong and Choi, Jungrak and Choi, Dong Yun and Yang, Quansan and Zhao, Hangbo and Bai, Wubin and Jang, Hokyung and Yu, Yongjoon and Lim,		

				Jaeman and Guo, Xu and Kim, Bong Hoon and Jeon, Seokwoo and Davies, Charles and Banks, Anthony and Sung, Hyung Jin and Huang, Yonggang and Park, Inkyu and Rogers, John A.		
Recent advances on 3D-bioprinted gelatin methacrylate hydrogels for tissue engineering in wound healing: A review of current applications and future prospects.	2024	International wound journal	1742-481X 1742-4801	Wang, Hongyu and Wan, Jiaming and Zhang, Zhiqiang and Hou, Ruixing	10.1111/iwj.14533	RAYYAN-INCLUSION: {"Asmaa"=>"Excluded", "Syafira"=>"Excluded"} | RAYYAN-EXCLUSION-REASONS: wrong publication type
Highly Sensitive and Wearable Liquid Metal-Based Pressure Sensor for Health Monitoring Applications: Integration of a 3D-Printed Microbump Array with the Microchannel.	2019	Advanced healthcare materials	2192-2659 2192-2640	Kim, Kyuyoung and Choi, Jungrak and Jeong, Yongrok and Cho, Incheol and Kim, Minseong and Kim, Seunghwan and Oh, Yongsuk and Park, Inkyu	10.1002/adhm.201900978	RAYYAN-INCLUSION: {"Asmaa"=>"Excluded", "Syafira"=>"Excluded"}
3D bioprinting of Salvianolic acid B-sodium alginate-gelatin skin scaffolds promotes diabetic wound repair via antioxidant, anti-inflammatory, and proangiogenic effects.	2024	Biomedicine & pharmacotherapy = Biomedecine & pharmacotherapie	1950-6007 0753-3322	Lihao, Qin and Tingting, Liu and Jiawei, Zhang and Yifei, Bai and Zheyu, Tang and Jingyan, Li and Tongqing, Xue and Zhongzhi, Jia	10.1016/j.biopha.2024.116168	RAYYAN-INCLUSION: {"Asmaa"=>"Excluded", "Syafira"=>"Included"} | RAYYAN-LABELS: no cell
Management of Acute Wounds - Expert Panel	2024	Advances in wound care	2162-1918	Olutoye, Oluyinka O. and Eriksson,	10.1089/wound.2023.0059	RAYYAN-INCLUSION: {"Asmaa"=>"Excluded", "Syafira"=>"Excluded"}

Consensus Statement.				Elof and Menchaca, Alicia D. and Kirsner, Robert S. and Tanaka, Rica and Schultz, Gregory and Weir, Dot and Wagner, Tracey and Renata, Fabia and Naik-Mathuria, Bindi and Liu, Paul and Ead, Karim J. and Adedayo, Temitope and Armstrong, David G. and McMullin, Neil and Balch Samora, Julie and Akingba, Ajibola G.		| USER-NOTES: {"Syafira"=>["acute wound"]}
A Wirelessly Controlled Smart Bandage with 3D-Printed Miniaturized Needle Arrays.	2020	Advanced functional materials	1616-301X 1616-3028	Derakhshandeh, Hossein and Aghabaglou, Fariba and McCarthy, Alec and Mostafavi, Azadeh and Wiseman, Chris and Bonick, Zack and Ghanavati, Ian and Harris, Seth and Kreikemeier-Bower, Craig and Basri, Seyed Masoud Moosavi and Rosenbohm, Jordan and Yang, Ruiguo and Mostafalu, Pooria and Orgill, Dennis and Tamayol, Ali	10.1002/adfm.201905544	RAYYAN-INCLUSION: {"Asmaa"=>"Excluded", "Syafira"=>"Excluded"}
Alginate Hydrogels with Embedded ZnO	2020	International journal of nanomedicine	1178-2013	Cleetus, Carol M. and Alvarez	10.2147/IJN.S255937	RAYYAN-INCLUSION: {"Asmaa"=>"Excluded", "Syafira"=>"Excluded"}

Nanoparticles for Wound Healing Therapy.			1176-9114	Primo, Fabian and Fregoso, Gisel and Lalitha Raveendran, Nivedita and Noveron, Juan C. and Spencer, Charles T. and Ramana, Chinatalapalle V. and Joddar, Binata		| RAYYAN-LABELS: no cell | USER-NOTES: {"Syafira"=>["not bionk"]}
Harnessing cytokine-induced killer cells to accelerate diabetic wound healing: an approach to regulating post-traumatic inflammation.	2024	Regenerative biomaterials	2056-3418 2056-3426	Yang, Yixi and Zhang, Cheng and Jiang, Yuan and He, Yijun and Cai, Jiawei and Liang, Lin and Chen, Zhaohuan and Pan, Sicheng and Hua, Chu and Wu, Keke and Wang, Le and Zhang, Zhiyong	10.1093/rb/rbad116	RAYYAN-INCLUSION: {"Asmaa"=>"Excluded", "Syafira"=>"Excluded"} | RAYYAN-LABELS: not 3D bioprint
Automatic quantitative analysis of structure parameters in the growth cycle of artificial skin using optical coherence tomography.	2021	Journal of biomedical optics	1560-2281 1083-3668	Zhao, Ruihang and Tang, Han and Xu, Chen and Ge, Yakun and Wang, Ling and Xu, Mingen	10.1117/1.JBO.26.9.095001	RAYYAN-INCLUSION: {"Asmaa"=>"Excluded", "Syafira"=>"Excluded"}
Incorporating a structural extracellular matrix gradient into a porcine urinary bladder matrix-based hydrogel dermal scaffold.	2021	Journal of biomedical materials research. Part A	1552-4965 1549-3296	Allbritton-King, Jules D. and Kimicata, Megan and Fisher, John P.	10.1002/jbm.a.37181	RAYYAN-INCLUSION: {"Asmaa"=>"Excluded", "Syafira"=>"Included"} | RAYYAN-LABELS: not 3D bioprint
Bacteriophage-cocktail hydrogel dressing to prevent multiple bacterial infections and heal diabetic ulcers in mice.	2024	Journal of biomedical materials research. Part A	1552-4965 1549-3296	Shiue, Sheng-Jie and Wu, Ming-Shun and Chiang, Yi-Hsien and Lin, Hsin-Yi	10.1002/jbm.a.37728	RAYYAN-INCLUSION: {"Asmaa"=>"Excluded", "Syafira"=>"Included"} | RAYYAN-LABELS: no access

Natural Polymer-Based Materials for Wound Healing Applications	2024	Advanced NanoBiomed Research	26999307 (ISSN)	Ho, T.T.-P. and Tran, H.A. and Doan, V.K. and Maitz, J. and Li, Z. and Wise, S.G. and Lim, K.S. and Rnjak-Kovacina, J.	10.1002/anbr.202300131	RAYYAN-INCLUSION: {"Asmaa"=>"Excluded", "Syafira"=>"Excluded"} | RAYYAN-EXCLUSION-REASONS: wrong publication type
Pharmaceutical applications of chitosan in skin regeneration: A review	2024	International Journal of Biological Macromolecules	01418130 (ISSN)	Wang, J. and Duan, X. and Zhong, D. and Zhang, M. and Li, J. and Hu, Z. and Han, F.	10.1016/j.ijbiomac.2023.129064	RAYYAN-INCLUSION: {"Asmaa"=>"Excluded", "Syafira"=>"Excluded"} | RAYYAN-EXCLUSION-REASONS: wrong publication type
Mesenchymal stromal cells in wound healing applications: role of the secretome, targeted delivery and impact on recessive dystrophic epidermolysis bullosa treatment	2021	Cytotherapy	14653249 (ISSN)	Riedl, J. and Popp, C. and Eide, C. and Ebens, C. and Tolar, J.	10.1016/j.jcyt.2021.06.004	AYYAN-INCLUSION: {"Asmaa"=>"Excluded", "Syafira"=>"Excluded"} | RAYYAN-EXCLUSION-REASONS: wrong publication type
3D-bioprinted double-crosslinked angiogenic alginate/chondroitin sulfate patch for diabetic wound healing	2023	International Journal of Biological Macromolecules	01418130 (ISSN)	Liao, W. and Duan, X. and Xie, F. and Zheng, D. and Yang, P. and Wang, X. and Hu, Z.	10.1016/j.ijbiomac.2023.123952	RAYYAN-INCLUSION: {"Asmaa"=>"Excluded", "Syafira"=>"Included", "Izzah Fadilah"=>"Excluded"} | RAYYAN-LABELS: no cell | USER-NOTES: {"Asmaa"=>["no encapsulated cells "]}
Tannic acid-loaded chitosan-RGD-alginate scaffolds for wound healing and skin regeneration	2023	Biomedical Materials (Bristol)	17486041 (ISSN)	Mndlovu, H. and du Toit, L.C. and Kumar, P. and Choonara, Y.E.	10.1088/1748-605X/acce88	RAYYAN-INCLUSION: {"Asmaa"=>"Excluded", "Syafira"=>"Included", "Izzah Fadilah"=>"Excluded"} | RAYYAN-LABELS: no cell,not 3D bioprint | USER-NOTES: {"Asmaa"=>["Not-3D Bioprinting Technologies, no cell, not chronic "]}
Bioactive glasses as biologically active materials for healing of skin wounds	2022	Bioactive Glasses and Glass-Ceramics: Fund. and Applic.	978-111972419-3 (ISBN); 978-1119724	Mehrabi, T. and Mesgar, A.S. and Mohammadi, Z.		RAYYAN-INCLUSION: {"Asmaa"=>"Excluded", "Syafira"=>"Excluded"} | RAYYAN-EXCLUSION-REASONS: wrong publication type

			51-3 (ISBN)			
Role of wound microbiome, strategies of microbiota delivery system and clinical management	2023	Advanced Drug Delivery Reviews	0169409X (ISSN)	Tang, Q. and Xue, N. and Ding, X. and Tsai, K.H.-Y. and Hew, J.J. and Jiang, R. and Huang, R. and Cheng, X. and Ding, X. and Yee Cheng, Y. and Chen, J. and Wang, Y.	10.1016/j.addr.2022.114671	RAYYAN-INCLUSION: {"Asmaa"=>"Excluded", "Syafira"=>"Excluded"} | RAYYAN-EXCLUSION-REASONS: wrong publication type
Therapy of Extensive Chronic Skin Defects after a Traumatic Injury Due to Microbial Contamination Using a Surface Implant Made of a Biocompatible Polycaprolactoneâ€"A Pilot Case Study	2022	Polymers	20734360 (ISSN)	Findrik BalogovÃ¡, A. and KoÅ¾Ã¡r, M. and StaroÅˆovÃ¡, R. and Schnitzer, M. and DancÃ¡kovÃ¡, G. and Å½ivÄ□Ã¡k, J. and HudÃ¡k, R.	10.3390/polym14235293	RAYYAN-INCLUSION: {"Asmaa"=>"Excluded", "Syafira"=>"Excluded"}
Quercus infectoria gall loaded patches for wound dressing: A comparison of fabrication methods	2022	Journal of Herbal Medicine	22108033 (ISSN)	AydÄ±n, S.T. and Demirhan, I. and ÅžengÃ¶r, M.	10.1016/j.hermed.2022.100605	RAYYAN-INCLUSION: {"Asmaa"=>"Excluded", "Syafira"=>"Excluded"} | RAYYAN-LABELS: fabrication only no biological
Exploring Skin Wound Healing Models and the Impact of Natural Lipids on the Healing Process	2024	International Journal of Molecular Sciences	16616596 (ISSN)	Choudhary, V. and Choudhary, M. and Bollag, W.B.	10.3390/ijms25073790	RAYYAN-INCLUSION: {"Asmaa"=>"Excluded", "Syafira"=>"Excluded"} | RAYYAN-EXCLUSION-REASONS: wrong publication type
Oxygen releasing patches based on carbohydrate polymer and protein hydrogels for diabetic wound healing: A review	2023	International Journal of Biological Macromolecules	01418130 (ISSN)	Al Mamun, A. and Ullah, A. and Chowdhury, M.E.H. and Marei, H.E. and Madappura, A.P. and Hassan, M. and Rizwan, M. and Gomes, V.G. and Amirfazli, A. and Hasan, A.	10.1016/j.ijbiomac.2023.126174	RAYYAN-INCLUSION: {"Asmaa"=>"Excluded", "Syafira"=>"Excluded"} | RAYYAN-EXCLUSION-REASONS: wrong publication type

3D-printed hydrogels based on amphiphilic chitosan derivative loaded with levofloxacin for wound healing applications	2024	International Journal of Polymeric Materials and Polymeric Biomaterials	00914037 (ISSN)	Lazaridou, M. and Moroni, S. and Klonos, P. and Kyritsis, A. and Bikiaris, D.N. and Lamprou, D.A.	10.1080/00914037.2024.2314610	RAYYAN-INCLUSION: {"Asmaa"=>"Excluded", "Syafira"=>"Excluded"} | RAYYAN-LABELS: not chronic ,fabrication only no biological
Three-dimensional shape-conformation performances of wound dressings tested in a robotic sacral pressure ulcer phantom	2021	International Wound Journal	17424801 (ISSN)	Lustig, A. and Gefen, A.	10.1111/iwj.13569	RAYYAN-INCLUSION: {"Asmaa"=>"Excluded", "Syafira"=>"Excluded"} | RAYYAN-LABELS: fabrication only no biological
Chronic wounds: pathological characteristics and their stem cell-based therapies	2023	Engineered Regeneration	26661381 (ISSN)	Wu, X. and Zhu, H. and Xu, Y. and Kong, B. and Tan, Q.	10.1016/j.engreg.2022.11.004	RAYYAN-INCLUSION: {"Asmaa"=>"Excluded", "Syafira"=>"Excluded"} | RAYYAN-EXCLUSION-REASONS: wrong publication type
Initial Clinical Experience with a Simple, Home System for Early Detection and Monitoring of Diabetic Foot Ulcers: The Foot Selfie	2023	Journal of Diabetes Science and Technology	19322968 (ISSN)	Swerdlow, M. and Shin, L. and Dâ€™Huyvetter, K. and Mack, W.J. and Armstrong, D.G.	10.1177/19322968211053348	RAYYAN-INCLUSION: {"Asmaa"=>"Excluded", "Syafira"=>"Excluded"} | RAYYAN-EXCLUSION-REASONS: background article
3D printed composite dressings loaded with human epidermal growth factor for potential chronic wound healing applications	2023	Journal of Drug Delivery Science and Technology	17732247 (ISSN)	Boateng, J.S. and Hafezi, F. and Tabriz, A.G. and Douroumis, D.	10.1016/j.jddst.2023.104684	RAYYAN-INCLUSION: {"Asmaa"=>"Excluded", "Syafira"=>"Included", "Izzah Fadilah"=>"Excluded"} | RAYYAN-LABELS: no cell | USER-NOTES: {"Asmaa"=>["no bioink , loaded with bioactive agents like epidermal growth factor (EGF), use cell seed for mtt "]}
Engineered artificial skins: Current construction strategies and applications	2023	Engineered Regeneration	26661381 (ISSN)	Xu, Y. and Wu, X. and Zhang, Y. and Yu, Y. and Gan, J. and Tan, Q.	10.1016/j.engreg.2023.09.001	RAYYAN-INCLUSION: {"Asmaa"=>"Excluded", "Syafira"=>"Excluded"} | RAYYAN-EXCLUSION-REASONS: wrong publication type
MR-compatible loading device for assessment of heel pad	2021	Medical Engineering and Physics	13504533 (ISSN)	Trebbi, A. and Perrier, A. and Bailet,	10.1016/j.medengphy.2021.11.006	RAYYAN-INCLUSION: {"Asmaa"=>"Excluded", "Syafira"=>"Excluded"} | RAYYAN-

internal tissue displacements under shearing load.				M. and Payan, Y.		EXCLUSION-REASONS: wrong publication type
Convergence of Biofabrication Technologies and Cell Therapies for Wound Healing	2022	Pharmaceutics	19994923 (ISSN)	Hosseini, M. and Dalley, A.J. and Shafiee, A.	10.3390/pharmaceutics14122749	RAYYAN-INCLUSION: {"Asmaa"=>"Excluded", "Syafira"=>"Excluded"} | RAYYAN-EXCLUSION-REASONS: wrong publication type
A Complete Sojorum on Thermosensitive Hydrogels for Wound Healing: Recent Developments and Ongoing Research	2024	Current Drug Therapy	15748855 (ISSN)	Markandeywar, T.S. and Singh, D. and Narang, R.K.	10.2174/1574885518666230505151446	RAYYAN-INCLUSION: {"Asmaa"=>"Excluded", "Syafira"=>"Excluded"} | RAYYAN-EXCLUSION-REASONS: wrong publication type
Comparison of two conchal formers for nonsurgical correction on Conchal Crus	2023	Laryngoscope Investigative Otolaryngology	23788038 (ISSN)	Zou, Q. and Zhao, S. and Wang, D. and Chen, P. and Yang, L. and Gao, M. and Liu, Y. and Zhao, C. and Li, S. and Yang, J.	10.1002/lio2.987	RAYYAN-INCLUSION: {"Asmaa"=>"Excluded", "Syafira"=>"Excluded"} | RAYYAN-EXCLUSION-REASONS: wrong publication type | USER-NOTES: {"Asmaa"=>["different topic"]}
Recent advances in 3D printed microneedles and their skin delivery application in the treatment of various diseases	2023	Journal of Drug Delivery Science and Technology	17732247 (ISSN)	Parhi, R.	10.1016/j.jddst.2023.104395	RAYYAN-INCLUSION: {"Asmaa"=>"Excluded", "Syafira"=>"Excluded"} | RAYYAN-EXCLUSION-REASONS: wrong publication type
Development of a Tacrolimus-loaded carboxymethyl chitosan scaffold as an effective 3D-printed wound dressing	2023	Journal of Drug Delivery Science and Technology	17732247 (ISSN)	Al-Hashmi, S. and Vakilian, S. and Jamshidi-adegani, F. and Al-Kindi, J. and Al-Fahdi, F. and Al-Hatmi, A.M.S. and Al-Jahdhami, H. and Anwar, M.U. and Al-Wahaibi, N. and Shalaby, A. and Al-Harrasi, A.	10.1016/j.jddst.2023.104707	RAYYAN-INCLUSION: {"Asmaa"=>"Excluded", "Syafira"=>"Excluded"} | USER-NOTES: {"Syafira"=>["Cell seeded on plate (2D) and on top of hydrogel (live dead). They mentioned it was a bioink but they dont encapsulate the cell in the formulation"], "Asmaa"=>["does not indicate the presence of living cells within the bioink."]}
Bioinspired Adaptable Indwelling Microneedles for	2023	Advanced Materials	09359648 (ISSN)	Zhang, X. and Gan, J. and Fan, L. and Luo, Z. and Zhao, Y.	10.1002/adma.202210903	RAYYAN-INCLUSION: {"Asmaa"=>"Excluded", "Syafira"=>"Included", "Izzah Fadilah"=>"Excluded"} |

Treatment of Diabetic Ulcers						RAYYAN-LABELS: no cell | USER-NOTES: {"Asmaa"=>["Does not meet the bioink criteria, as the encapsulated substance is exosomes rather than living cells."]}
Recent advances in 3D printing for wound healing: A systematic review	2022	Journal of Drug Delivery Science and Technology	17732247 (ISSN)	Tabriz, A.G. and Douroumis, D.	10.1016/j.jddst.2022.103564	RAYYAN-INCLUSION: {"Asmaa"=>"Excluded", "Syafira"=>"Excluded"} | RAYYAN-EXCLUSION-REASONS: wrong publication type
Innovations and Advances in Wound Healing, Third Edition	2023	Innovations and Advances in Wound Healing, Third Edition	978-981199805-8 (ISBN); 978-981199804-1 (ISBN)	Han, S.-K.		RAYYAN-INCLUSION: {"Asmaa"=>"Excluded", "Syafira"=>"Excluded"} | RAYYAN-EXCLUSION-REASONS: wrong publication type
Threads of hope: Harnessing nanofibres-based treatment strategies for diabetic foot ulcers	2024	Journal of Drug Delivery Science and Technology	17732247 (ISSN)	Vyas, G. and Karpe, S. and Gupta, K. and Lad, S. and Kaur, C. and Sharma, S. and Singh, G. and Saini, S. and Kumar, R.	10.1016/j.jddst.2023.105225	RAYYAN-INCLUSION: {"Asmaa"=>"Excluded", "Syafira"=>"Excluded"} | RAYYAN-EXCLUSION-REASONS: wrong publication type
Skin substitutes as treatment for chronic wounds: current and future directions	2023	Frontiers in Medicine	2296858X (ISSN)	Vecin, N.M. and Kirsner, R.S.	10.3389/fmed.2023.1154567	RAYYAN-INCLUSION: {"Asmaa"=>"Excluded", "Syafira"=>"Excluded"} | RAYYAN-EXCLUSION-REASONS: wrong publication type
Injectable and Microporous Microgel-Fiber Granular Hydrogel Loaded with Bioglass and siRNA for Promoting Diabetic Wound Healing	2023	Small	16136810 (ISSN)	Li, Y. and Song, W. and Kong, L. and He, Y. and Li, H.	10.1002/smll.202309599	RAYYAN-INCLUSION: {"Asmaa"=>"Excluded", "Syafira"=>"Excluded"} | RAYYAN-LABELS: not 3D bioprint
In vivo printing of growth factor-eluting adhesive scaffolds improves wound healing	2022	Bioactive Materials	2452199X (ISSN)	Nuutila, K. and Samandari, M. and Endo, Y. and Zhang, Y. and Quint, J. and Schmidt, T.A. and Tamayol,	10.1016/j.bioactmat.2021.06.030	RAYYAN-INCLUSION: {"Asmaa"=>"Excluded", "Syafira"=>"Excluded"} | RAYYAN-LABELS: no cell | USER-NOTES: {"Syafira"=>["this study incorporated VEGF into GelMA to develop bioink. Not cell"]}

				A. and Sinha, I.		
Role of microbiome, microenvironment and novel drug delivery system in the wound healing	2023	Advanced Drug Delivery Reviews	0169409X (ISSN)	Luo, G. and Maitz, P.K.M. and Wang, Y.	10.1016/j.addr.2023.114873	RAYYAN-INCLUSION: {"Asmaa"=>"Excluded", "Syafira"=>"Excluded"} | RAYYAN-EXCLUSION-REASONS: wrong publication type
Bioink hydrogel from fish scale gelatin blended with alginate for 3D-bioprinting application	2022	Journal of Food Processing and Preservation	01458892 (ISSN)	Boonyagul, S. and Pukasamsombut, D. and Pengpanich, S. and Toobunterng, T. and Pasanaphong, K. and Sathirapongsasuti, N. and Tawonsawatruk, T. and Wangtueai, S. and Tanadchangsaeng, N.	10.1111/jfpp.15864	RAYYAN-INCLUSION: {"Asmaa"=>"Excluded", "Syafira"=>"Included", "Izzah Fadilah"=>"Included"} | RAYYAN-LABELS: fabrication only no biological | USER-NOTES: {"Syafira"=>["This study performed bioink (cell line encapsulation with the bioink). The characterization done based on wound healing properties needed. However, not highlighted on chronic wound healing. But, potentially use for chronic wound healing."]}
Portable Skin-injury Monitoring Device for Early Diagnosis of Pressure Ulcers	2022	Sensors and Materials	09144935 (ISSN)	Zhang, L. and Takashi, E. and Lu, J. and Kamijo, A. and Kitayama, A.	10.18494/SAM3601	RAYYAN-INCLUSION: {"Asmaa"=>"Excluded", "Syafira"=>"Excluded"} | RAYYAN-EXCLUSION-REASONS: wrong publication type
Chronic Wound Healing Models	2023	ACS Pharmacology and Translational Science	25759108 (ISSN)	Flynn, K. and Mahmoud, N.N. and Sharifi, S. and Gould, L.J. and Mahmoudi, M.	10.1021/acsptsci.3c00030	RAYYAN-INCLUSION: {"Asmaa"=>"Excluded", "Syafira"=>"Excluded"} | RAYYAN-EXCLUSION-REASONS: wrong publication type
Bio-based polymers containing traditional medicinal fillers for wound healing applications â€" An evaluation of neoteric development and future perspectives	2023	Biotechnology Journal	18606768 (ISSN)	Hema, S. and Unni, V.V. and Niranjan, B. and Chandran, S. and Sambhudevan, S.	10.1002/biot.202300006	RAYYAN-INCLUSION: {"Asmaa"=>"Excluded", "Syafira"=>"Excluded"} | RAYYAN-EXCLUSION-REASONS: wrong publication type
Evaluation of the optimum	2022	Proceedings of the Annual	1557170X	Bayram, M.B. and	10.1109/EMBC48229.2022.9871428	RAYYAN-INCLUSION: {"Asmaa"=>"Excluded",

positioning for a multi-use and wearable pressure ulcer sensor		International Conference of the IEEE Engineering in Medicine and Biology Society, EMBS	(ISSN); 978-172812782-8 (ISBN)	Kaykayoglu, C.A.		"Syafira"=>"Excluded"} | RAYYAN-EXCLUSION-REASONS: wrong publication type
Dermal Fibroblast Heterogeneity and Its Contribution to the Skin Repair and Regeneration	2022	Advances in Wound Care	21621918 (ISSN)	Xue, M. and Zhao, R. and March, L. and Jackson, C.	10.1089/wound.2020.1287	RAYYAN-INCLUSION: {"Asmaa"=>"Excluded", "Syafira"=>"Excluded"} | RAYYAN-EXCLUSION-REASONS: wrong publication type
Sponge-Like Macroporous Hydrogel with Antibacterial and ROS Scavenging Capabilities for Diabetic Wound Regeneration	2022	Advanced Healthcare Materials	21922640 (ISSN)	Wei, C. and Tang, P. and Tang, Y. and Liu, L. and Lu, X. and Yang, K. and Wang, Q. and Feng, W. and Shubhra, Q.T.H. and Wang, Z. and Zhang, H.	10.1002/adhm.202200717	RAYYAN-INCLUSION: {"Asmaa"=>"Excluded", "Syafira"=>"Included", "Izzah Fadilah"=>"Excluded"} | RAYYAN-LABELS: not 3D bioprint | USER-NOTES: {"Asmaa"=>["not bioink, not 3d bioprinting ", "no access to full text "]}
Application of 3D printing & 3D bioprinting for promoting cutaneous wound regeneration	2022	Bioprinting	24058866 (ISSN)	Sun, Y. and Juncos Bombin, A.D. and Boyd, P. and Dunne, N. and McCarthy, H.O.	10.1016/j.bprint.2022.e00230	RAYYAN-INCLUSION: {"Asmaa"=>"Excluded", "Syafira"=>"Excluded"} | RAYYAN-EXCLUSION-REASONS: wrong publication type
Triple-layered core-shell fiber dressings with enduring platelet conservation and sustained growth factor release abilities for chronic wound healing	2024	Regenerative Biomaterials	20563418 (ISSN)	Lai, S. and Wu, T. and Shi, C. and Wang, X. and Liu, P. and Wang, L. and Yu, H.	10.1093/rb/rbae034	RAYYAN-INCLUSION: {"Asmaa"=>"Excluded", "Syafira"=>"Maybe"} | RAYYAN-LABELS: no cell | USER-NOTES: {"Asmaa"=>["include. The bioink includes PRP, which contains platelets (a component of living cells)."]}
Membranous extracellular matrix-based scaffolds for skin wound healing	2021	Pharmaceutics	19994923 (ISSN)	Da, L.-C. and Huang, Y.-Z. and Xie, H.-Q. and Zheng, B.-H. and Huang, Y.-C. and Du, S.-R.	10.3390/pharmaceutics13111796	RAYYAN-INCLUSION: {"Asmaa"=>"Excluded", "Syafira"=>"Excluded"} | RAYYAN-EXCLUSION-REASONS: wrong publication type
Skin regeneration, repair, and reconstruction: present and future	2022	European Surgery - Acta Chirurgica Austriaca	16828631 (ISSN)	Kamolz, L.-P. and Kotzbeck, P. and Schintler, M. and Spendel, S.	10.1007/s10353-022-00757-9	RAYYAN-INCLUSION: {"Asmaa"=>"Excluded", "Syafira"=>"Excluded"} | RAYYAN-EXCLUSION-REASONS: wrong publication type

Bioactive wound dressings for the management of chronic non healing ulcers (CNHU) â€“ A review of clinical and translational studies	2022	Materialia	25891529 (ISSN)	Sarkar, S. and Poundarik, A.A.	10.1016/j.mtla.2021.101269	RAYYAN-EXCLUSION-REASONS: wrong publication type
Skin wound healing: The critical role of angiogenesis	2022	Biomaterials for Vasculogenesis and Angiogenesis	978-012821867-9 (ISBN); 978-012821868-6 (ISBN)	Nazarnezhad, S. and Kargozar, S. and Hamblin, M.R.		RAYYAN-INCLUSION: {"Asmaa"=>"Excluded", "Syafira"=>"Excluded"} | RAYYAN-EXCLUSION-REASONS: wrong publication type
Tissue Engineering-Based Strategies for Diabetic Foot Ulcer Management	2023	Advances in Wound Care	21621918 (ISSN)	Chiu, A. and Sharma, D. and Zhao, F.	10.1089/wound.2021.0081	RAYYAN-INCLUSION: {"Asmaa"=>"Excluded", "Syafira"=>"Excluded"} | RAYYAN-EXCLUSION-REASONS: wrong publication type
Graphical Abstract TOC	2023	Journal of Drug Delivery Science and Technology	17732247 (ISSN)		10.1016/S1773-2247(23)00987-5	RAYYAN-INCLUSION: {"Asmaa"=>"Excluded", "Syafira"=>"Excluded"}
Current status and progress in research on dressing management for diabetic foot ulcer	2023	Frontiers in Endocrinology	16642392 (ISSN)	Jiang, P. and Li, Q. and Luo, Y. and Luo, F. and Che, Q. and Lu, Z. and Yang, S. and Yang, Y. and Chen, X. and Cai, Y.	10.3389/fendo.2023.1221705	RAYYAN-INCLUSION: {"Asmaa"=>"Excluded", "Syafira"=>"Excluded"} | RAYYAN-EXCLUSION-REASONS: wrong publication type
Current Advances in Wound Healing and Regenerative Medicine	2024	Current Stem Cell Research and Therapy	1574888X (ISSN)	Fani, N. and Moradi, M. and Zavari, R. and Parvizpour, F. and Soltani, A. and Arabpour, Z. and Jafarian, A.	10.2174/1574888X18666230301140659	RAYYAN-INCLUSION: {"Asmaa"=>"Excluded", "Syafira"=>"Excluded"} | RAYYAN-EXCLUSION-REASONS: wrong publication type
New insights into biomaterials for wound dressings and care: Challenges and trends	2024	Progress in Organic Coatings	03009440 (ISSN)	Moreira, T.D. and Martins, V.B. and da Silva JÃºnior, A.H. and Sayer, C. and de AraÃºjo, P.H.H. and Immich, A.P.S.	10.1016/j.porgcoat.2023.108118	RAYYAN-INCLUSION: {"Asmaa"=>"Excluded", "Syafira"=>"Excluded"} | RAYYAN-EXCLUSION-REASONS: wrong publication type
Bioprinting technology for the management of diabetic foot	2023	International Journal of Bioprinting	24248002 (ISSN)	Xu, F. and Rui, S. and Yang, C. and Jiang, X. and	10.36922/IJB.0142	RAYYAN-INCLUSION: {"Asmaa"=>"Excluded", "Syafira"=>"Excluded"} | RAYYAN-

disease: Emerging applications, challenges, and prospects				Wu, W. and Tang, X. and Armstrong, D.G. and Ma, Y. and Deng, W.		EXCLUSION-REASONS: wrong publication type
In Situ Bioprinting of Autologous Skin Cells Accelerates Wound Healing of Extensive Excisional Full-Thickness Wounds	2019	Scientific Reports	20452322 (ISSN)	Albanna, M. and Binder, K.W. and Murphy, S.V. and Kim, J. and Qasem, S.A. and Zhao, W. and Tan, J. and El-Amin, I.B. and Dice, D.D. and Marco, J. and Green, J. and Xu, T. and Skardal, A. and Holmes, J.H. and Jackson, J.D. and Atala, A. and Yoo, J.J.	10.1038/s41598-018-38366-w	RAYYAN-INCLUSION: {"Asmaa"=>"Included", "Syafira"=>"Excluded"} | USER-NOTES: {"Asmaa"=>["Good one, not really indicate the chronic wound but it shows chronic inflammation during the treatment"]}
Polysaccharide thin solid films for analgesic drug delivery and growth of human skin cells	2019	Frontiers in Chemistry	22962646 (ISSN)	Maver, T. and Mohan, T. and GradiÅ¡nik, L. and FinÅ¡gar, M. and Kleinschek, K.S. and Maver, U.	10.3389/fchem.2019.00217	RAYYAN-INCLUSION: {"Asmaa"=>"Excluded", "Syafira"=>"Excluded"} | RAYYAN-LABELS: not 3D bioprint
Polysaccharide-based materials created by physical processes: From preparation to biomedical applications	2021	Pharmaceutics	19994923 (ISSN)	Souza, P.R. and de Oliveira, A.C. and Vilsinski, B.H. and Kipper, M.J. and Martins, A.F.	10.3390/pharmaceutics13050621	RAYYAN-INCLUSION: {"Asmaa"=>"Excluded", "Syafira"=>"Excluded"} | RAYYAN-EXCLUSION-REASONS: wrong publication type
Advances in generation of three-dimensional skin equivalents: pre-clinical studies to clinical therapies	2021	Cytotherapy	14653249 (ISSN)	Choudhury, S. and Das, A.	10.1016/j.jcyt.2020.10.001	RAYYAN-INCLUSION: {"Asmaa"=>"Excluded", "Syafira"=>"Excluded"} | RAYYAN-EXCLUSION-REASONS: wrong publication type
Current trends in advanced alginate-based wound dressings for chronic wounds	2021	Journal of Personalized Medicine	20754426 (ISSN)	Barbu, A. and Neamtu, B. and ZÄƒhan, M. and Iancu, G.M. and Bacila, C. and MireÈ™an, V.	10.3390/jpm11090890	RAYYAN-INCLUSION: {"Asmaa"=>"Excluded", "Syafira"=>"Excluded"} | RAYYAN-EXCLUSION-REASONS: wrong publication type

Honey loaded silk fibroin 3D porous scaffold facilitates homeostatic full-thickness wound healing	2020	Materialia	25891529 (ISSN)	Rajput, M. and Mandal, M. and Anura, A. and Mukhopadhyay, A. and Subramanian, B. and Paul, R.R. and Chatterjee, J.	10.1016/j.mtla.2020.100703	RAYYAN-INCLUSION: {"Asmaa"=>"Excluded", "Syafira"=>"Excluded"} | RAYYAN-LABELS: no cell,fabrication only no biological
Advances in skin regeneration and reconstruction	2020	Front. Stem Cell Regen. Med. Res.	24679593 (ISSN)	Rosca, A.-M. and Tutuianu, R. and Titorencu, I.		RAYYAN-INCLUSION: {"Asmaa"=>"Excluded", "Syafira"=>"Excluded"} | RAYYAN-EXCLUSION-REASONS: wrong publication type
A skin-inspired 3D bilayer scaffold enhances granulation tissue formation and anti-infection for diabetic wound healing	2019	Journal of Materials Chemistry B	20507518 (ISSN)	Wan, W. and Cai, F. and Huang, J. and Chen, S. and Liao, Q.	10.1039/c8tb03341b	RAYYAN-INCLUSION: {"Asmaa"=>"Excluded", "Syafira"=>"Excluded"} | RAYYAN-LABELS: no cell | USER-NOTES: {"Syafira"=>["not bioink"]}
Active agents loaded extracellular matrix mimetic electrospun membranes for wound healing applications	2021	Journal of Drug Delivery Science and Technology	17732247 (ISSN)	Kalva, S.N. and Augustine, R. and Al Mamun, A. and Dalvi, Y.B. and Vijay, N. and Hasan, A.	10.1016/j.jddst.2021.102500	RAYYAN-INCLUSION: {"Asmaa"=>"Excluded", "Syafira"=>"Excluded"} | RAYYAN-EXCLUSION-REASONS: wrong publication type
A concise review on tissue engineered artificial skin grafts for chronic wound treatment: Can we reconstruct functional skin tissue in vitro?	2020	Cells	20734409 (ISSN)	Przekora, A.	10.3390/cells9071622	RAYYAN-INCLUSION: {"Asmaa"=>"Excluded", "Syafira"=>"Excluded"} | RAYYAN-EXCLUSION-REASONS: wrong publication type
The successful use of a bespoke OssDsign cranial plate to reconstruct an occipital defect following excision of a recurrent epithelioid sarcoma	2020	JPRAS Open	23525878 (ISSN)	Bloom, O. and Goddard, N. and Yannoulias, B. and Eccles, S.	10.1016/j.jpra.2020.01.002	RAYYAN-INCLUSION: {"Asmaa"=>"Excluded", "Syafira"=>"Excluded"} | RAYYAN-EXCLUSION-REASONS: wrong publication type
Design of Novel 3D-Scaffold as a Potential Material to Induct	2020	Fibers and Polymers	12299197 (ISSN)	Aghmiuni, A.I. and Baei, M.S. and Keshel, S.H.	10.1007/s12221-020-9402-1	RAYYAN-INCLUSION: {"Asmaa"=>"Excluded", "Syafira"=>"Included", "Izzah Fadilah"=>"Excluded"} |

Epidermal-Dermal Keratinocytes of Human-Adipose-Derived Stem Cells and Promote Fibroblast Cells Proliferation for Skin Regeneration				and Khiyavi, A.A.		RAYYAN-LABELS: not 3D bioprint | USER-NOTES: {"Asmaa"=>["full text screen: no bioink , There is no mention of using a 3D bioprinter for creating the scaffolds."]}
Method for improving skin color accuracy of three-dimensional printed training models for early pressure ulcer recognition	2019	Innovations and Emerg. Technologies in Wound Care	978-012815028-3 (ISBN); 978-012815029-0 (ISBN)	Krieger, L.W.M.M. and Ridge, T. and Demers, A. and Vishwanath, K. and Sparks, J.L.		RAYYAN-INCLUSION: {"Asmaa"=>"Excluded", "Syafira"=>"Excluded"}
Life Sciences Discovery and Technology Highlights	2019	SLAS Technology	24726303 (ISSN)	Yeo, D. and Murthy, T.	10.1177/2472630319862538	RAYYAN-INCLUSION: {"Asmaa"=>"Excluded", "Syafira"=>"Excluded"} | RAYYAN-EXCLUSION-REASONS: wrong publication type
Skin Stem Cells, Their Niche and Tissue Engineering Approach for Skin Regeneration	2020	Adv. Exp. Med. Biol.	00652598 (ISSN)	Ã‡ankirili, N.K. and Altundag, O. and Ã‡elebi-Saltik, B.		RAYYAN-INCLUSION: {"Asmaa"=>"Excluded", "Syafira"=>"Excluded"} | RAYYAN-EXCLUSION-REASONS: wrong publication type
Phantom testing of the sensitivity and precision of a sub-epidermal moisture scanner	2019	International Wound Journal	17424801 (ISSN)	Peko Cohen, L. and Gefen, A.	10.1111/iwj.13132	RAYYAN-INCLUSION: {"Asmaa"=>"Excluded", "Syafira"=>"Excluded"}
3D printed chitosan dressing crosslinked with genipin for potential healing of chronic wounds	2019	International Journal of Pharmaceutics	03785173 (ISSN)	Hafezi, F. and Scoutaris, N. and Douroumis, D. and Boateng, J.	10.1016/j.ijpharm.2019.02.020	RAYYAN-INCLUSION: {"Asmaa"=>"Excluded", "Syafira"=>"Maybe"} | RAYYAN-LABELS: no cell | USER-NOTES: {"Asmaa"=>["full text screen: no bioink "]}
Advanced drug delivery systems and artificial skin grafts for skin wound healing	2019	Advanced Drug Delivery Reviews	0169409X (ISSN)	Kim, H.S. and Sun, X. and Lee, J.-H. and Kim, H.-W. and Fu, X. and Leong, K.W.	10.1016/j.addr.2018.12.014	RAYYAN-INCLUSION: {"Asmaa"=>"Excluded", "Syafira"=>"Excluded"} | RAYYAN-EXCLUSION-REASONS: wrong publication type
3D-Printed Functional Hydrogel by DNA-Induced	2023	ADVANCED SCIENCE	2198-3844	Kim, N and Lee, H and Han, G and Kang, M and	10.1002/advs.202300816	RAYYAN-INCLUSION: {"Asmaa"=>"Excluded", "Syafira"=>"Included", "Izzah

Biomineralization for Accelerated Diabetic Wound Healing				Park, S and Kim, DE and Lee, M and Kim, MJ and Na, Y and Oh, S and Bang, SJ and Jang, TS and Kim, HE and Park, J and Shin, SR and Jung, HD		Fadilah"=>"Excluded"} | RAYYAN-LABELS: no cell | USER-NOTES: {"Asmaa"=>["no bioink"]}
Elastolytic-sensitive 3D-printed chitosan scaffold for wound healing applications	2021	MRS COMMUNICATIONS	2159-6859	Catanzano, O and Elviri, L and Bergonzi, C and Bianchera, A and Bettini, R and Bandiera, A	10.1557/s43579-021-00124-x	RAYYAN-INCLUSION: {"Asmaa"=>"Excluded", "Syafira"=>"Included", "Izzah Fadilah"=>"Excluded"} | RAYYAN-LABELS: no cell | USER-NOTES: {"Asmaa"=>["no cell encapsulated as bioink "]}
3D Bioprinting and Its Role in a Wound Healing Renaissance	2023	ADVANCED MATERIALS TECHNOLOGIES	2365-709X	Tanfani, JD and Monpara, JD and Jonnalagadda, S	10.1002/admt.202300411	RAYYAN-INCLUSION: {"Asmaa"=>"Excluded", "Syafira"=>"Excluded"} | RAYYAN-EXCLUSION-REASONS: wrong publication type
Bactericidal activity of 3D-printed hydrogel dressing loaded with gallium maltolate	2019	APL BIOENGINEERING	2473-2877	Cereceres, S and Lan, ZY and Bryan, L and Whitely, M and Wilems, T and Greer, H and Alexander, ER and Taylor, RJ and Bernstein, L and Cohen, N and Whitfield-Cargile, C and Cosgriff-Hernandez, E	10.1063/1.5088801	RAYYAN-INCLUSION: {"Asmaa"=>"Excluded", "Syafira"=>"Included", "Izzah Fadilah"=>"Excluded"} | RAYYAN-LABELS: no cell | USER-NOTES: {"Asmaa"=>["The study does not involve bioinks with living cells. The focus is on hydrogel dressings with gallium maltolate for antimicrobial purposes.\n"]}
3D Bioprinting Constructs to Facilitate Skin Regeneration	2022	ADVANCED FUNCTIONAL MATERIALS	1616-301X	Daikuara, LY and Chen, XF and Yue, ZL and Skropeta, D and Wood, FM and Fear, MW and Wallace, GG	10.1002/adfm.202105080	RAYYAN-INCLUSION: {"Asmaa"=>"Excluded", "Syafira"=>"Excluded"} | RAYYAN-EXCLUSION-REASONS: wrong publication type
Curcumin-incorporated 3D bioprinting gelatin methacryloyl hydrogel reduces	2022	BURNS & TRAUMA	2321-3868	Xia, SZ and Weng, TT and Jin, RH and Yang, M and Yu, MR and Zhang,	10.1093/burnst/tkac001	RAYYAN-INCLUSION: {"Asmaa"=>"Included", "Syafira"=>"Included"}

reactive oxygen species-induced adipose-derived stem cell apoptosis and improves implanting survival in diabetic wounds				W and Wang, XG and Han, CM		
Carboxymethyl cellulose-based materials for infection control and wound healing: A review	2020	INTERNATIONAL JOURNAL OF BIOLOGICAL MACROMOLECULES	0141-8130	Kanikireddy, V and Varaprasad, K and Jayaramudu, T and Karthikeyan, C and Sadiku, R	10.1016/j.ijbiomac.2020.07.160	RAYYAN-INCLUSION: {"Asmaa"=>"Excluded", "Syafira"=>"Excluded"} | RAYYAN-EXCLUSION-REASONS: wrong publication type
Applications of hydrogel-based delivery systems in wound care and treatment: An up-to-date review	2022	POLYMERS FOR ADVANCED TECHNOLOGIES	1042-7147	Divyashri, G and Badhe, RV and Sadanandan, B and Vijayalakshmi, V and Kumari, M and Ashrit, P and Bijukumar, D and Mathew, MT and Shetty, K and Raghu, AV	10.1002/pat.5661	RAYYAN-INCLUSION: {"Asmaa"=>"Excluded", "Syafira"=>"Excluded"} | RAYYAN-EXCLUSION-REASONS: wrong publication type
Bioengineered Skin Substitutes: Advances and Future Trends	2021	APPLIED SCIENCES-BASEL	2076-3417	Tavakoli, S and Klar, AS	10.3390/app11041493	RAYYAN-INCLUSION: {"Asmaa"=>"Excluded", "Syafira"=>"Excluded"} | RAYYAN-EXCLUSION-REASONS: wrong publication type
Chasing the Paradigm: Clinical Translation of 25 Years of Tissue Engineering	2019	TISSUE ENGINEERING PART A	1937-3341	Hoffman, T and Khademhosseini, A and Langer, R	10.1089/ten.tea.2019.0032	RAYYAN-INCLUSION: {"Asmaa"=>"Excluded", "Syafira"=>"Excluded"} | RAYYAN-EXCLUSION-REASONS: wrong publication type
Therapeutic strategies for skin regeneration based on biomedical substitutes	2019	JOURNAL OF THE EUROPEAN ACADEMY OF DERMATOLOGY AND VENEREOLOGY	0926-9959	Chocarro-Wrona, C and LÃ³pez-Ruiz, E and PerÃ¡n, M and GÃ¡lvez-MartÃn, P and Marchal, JA	10.1111/jdv.15391	RAYYAN-INCLUSION: {"Asmaa"=>"Excluded", "Syafira"=>"Excluded"} | RAYYAN-EXCLUSION-REASONS: wrong publication type
Engineering of biocompatible hybrid gelatin-PVA bioink for potential chronic wound treatment	2022	TISSUE ENGINEERING PART A	1937-3341	Masri, S and Maarof, M and Duman, F and Fauzi, MBM		RAYYAN-INCLUSION: {"Asmaa"=>"Included", "Syafira"=>"Included", "Izzah Fadilah"=>"Included"} | USER-NOTES:

						{"Asmaa"=>["does not mention the inclusion of living cells within the bioink. It focuses on the characterization and preparation of the bioink, physicochemical analysis, and cell-bioink interaction tests, but does not confirm the presence of living cells in the bioink."]}
A hydrogel wound dressing ideally designed for chronic wound care	2023	MATTER	2590-2393	Li, Y and Hao, DF and Feng, G and Xu, FJ	10.1016/j.matt.2023.03.006	RAYYAN-INCLUSION: {"Asmaa"=>"Excluded", "Syafira"=>"Excluded"} | RAYYAN-EXCLUSION-REASONS: wrong publication type
Amnion-derived hydrogels as a versatile platform for regenerative therapy: from lab to market	2024	FRONTIERS IN BIOENGINEERING AND BIOTECHNOLOGY	2296-4185	Kafili, G and Niknejad, H and Tamjid, E and Simchi, A	10.3389/fbioe.2024.1358977	RAYYAN-INCLUSION: {"Asmaa"=>"Excluded", "Syafira"=>"Excluded"} | RAYYAN-EXCLUSION-REASONS: wrong publication type

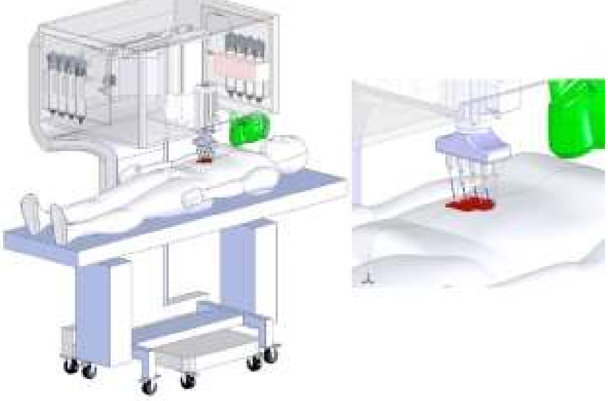

Figure S1. Schematic showing the scale, design, and components of the skin bioprinter. Adapted from Albanna et al. (2019)

Preferred Reporting Items for Systematic reviews and Meta-Analyses extension for Scoping Reviews (PRISMA-ScR) Checklist

SECTION	ITEM	PRISMA-ScR CHECKLIST ITEM	REPORTED ON PAGE #
**TITLE**			
Title	1	Application of 3D Printed Bioinks in Chronic Wound Healing: A Scoping Review	Page 1
**ABSTRACT**			
Structured summary	2	The abstract provides a clear structured summary with background, objectives, eligibility criteria, sources of evidence, charting methods, results, and conclusions.	Page 1
**INTRODUCTION**			
Rationale	3	The introduction outlines the clinical challenges of chronic wounds and the potential of 3D bioprinting in this context, justifying the need for a scoping review.	Page 2
Objectives	4	The objectives are clearly stated, focusing on evaluating the applications, methodologies, and effectiveness of 3D printed bioinks in chronic wound healing.	Page 6
**METHODS**			
Protocol and registration	5	https://doi.org/10.17605/OSF.IO/DP5Z6	Supplementary
Eligibility criteria	6	The criteria are well defined, focusing on original research articles involving 3D bioprinting technologies and bioinks containing living cells for chronic wound management.	Page 7
Information sources*	7	Comprehensive searches were conducted in PubMed, Scopus, and Web of Science databases, covering literature from 2019 to 2024.	Page 7
Search	8	The search strategy is detailed, including specific keywords and Boolean operators.	Page 7
Selection of sources of evidence†	9	The selection process is described, with independent screening by two reviewers and resolution of disagreements by a third reviewer if necessary.	Page 7

SECTION	ITEM	PRISMA-ScR CHECKLIST ITEM	REPORTED ON PAGE #
Data charting process‡	10	Data extraction was performed and verified by a second reviewer to ensure accuracy and alignment with the research questions.	Page 8
Data items	11	The data extracted included publication details, study design, types of 3D bioprinting techniques, materials and cells used, and specific outcomes related to chronic wound healing.	Page 7-8
Critical appraisal of individual sources of evidence§	12	If done, provide a rationale for conducting a critical appraisal of included sources of evidence; describe the methods used and how this information was used in any data synthesis (if appropriate).	Not applicable
Synthesis of results	13	The synthesis of results is described, focusing on qualitative synthesis and thematic analysis of the included studies.	Page 8
**RESULTS**			
Selection of sources of evidence	14	A PRISMA flow diagram is provided to illustrate the selection process, including the number of records identified, screened, and excluded.	Page 8-9
Characteristics of sources of evidence	15	Characteristics of the included studies, such as 3D bioprinting techniques, bioink compositions, and specific outcomes, are summarized.	Page 9-10
Critical appraisal within sources of evidence	16	If done, present data on critical appraisal of included sources of evidence (see item 12).	Not applicable
Results of individual sources of evidence	17	Data from individual sources are summarized in tables and described narratively, focusing on the diversity of bioink compositions and their effectiveness.	Page 10-22
Synthesis of results	18	The synthesis highlights the variation in study designs, materials, and outcomes, emphasizing the need for standardization and further research.	Page 10-22
**DISCUSSION**			
Summary of evidence	19	The discussion provides an overview of the advancements and challenges in using 3D printed bioinks for chronic wound healing, linking findings to the review objectives.	Page 22-24

SECTION	ITEM	PRISMA-ScR CHECKLIST ITEM	REPORTED ON PAGE #
Limitations	20	The limitations of the review are discussed, including the small number of studies, variability in methods, and exclusion of non-English articles.	Page 24-25
Conclusions	21	The conclusion provides a clear interpretation of the results, highlighting the potential for 3D bioprinting in chronic wound care and suggesting areas for future research.	Page 25
**FUNDING**			
Funding	22	Funding sources are acknowledged, noting that the research was supported by Universiti Kebangsaan Malaysia under the Dana Impak Perdana 2.0 grant.	Page 26

JBI = Joanna Briggs Institute; PRISMA-ScR = Preferred Reporting Items for Systematic reviews and Meta-Analyses extension for Scoping Reviews.

* Where *sources of evidence* (see second footnote) are compiled from, such as bibliographic databases, social media platforms, and Web sites.

† A more inclusive/heterogeneous term used to account for the different types of evidence or data sources (e.g., quantitative and/or qualitative research, expert opinion, and policy documents) that may be eligible in a scoping review as opposed to only studies. This is not to be confused with *information sources* (see first footnote).

‡ The frameworks by Arksey and O'Malley (6) and Levac and colleagues (7) and the JBI guidance (4, 5) refer to the process of data extraction in a scoping review as data charting.

§ The process of systematically examining research evidence to assess its validity, results, and relevance before using it to inform a decision. This term is used for items 12 and 19 instead of "risk of bias" (which is more applicable to systematic reviews of interventions) to include and acknowledge the various sources of evidence that may be used in a scoping review (e.g., quantitative and/or qualitative research, expert opinion, and policy document).

From: Tricco AC, Lillie E, Zarin W, O'Brien KK, Colquhoun H, Levac D, et al. PRISMA Extension for Scoping Reviews (PRISMAScR): Checklist and Explanation. Ann Intern Med. 2018;169:467–473. doi: 10.7326/M18-0850.
